# Sex disparities in the associations between accelerometer-measured movement behaviors and subsequent thromboembolism risk in cancer patients

**DOI:** 10.1186/s13293-026-00867-z

**Published:** 2026-03-02

**Authors:** Xiao Huang, Darui Gao, Wenya Zhang, Mengmeng Ji, Yang Pan, Yanyu Zhang, Yiwen Dai, Anshi Wu, Fanfan Zheng, Wuxiang Xie

**Affiliations:** 1 Clinical Research Institute, Institute of Advanced Clinical Medicine, Peking University, No. 38 Xueyuan Road, Haidian District, 100191 Beijing, China; 2 Department of Anesthesiology, Beijing Chao-Yang Hospital, Capital Medical University, No. 8, South Workers Stadium Road, Chaoyang District, Beijing, 100020 China; 3 Key Laboratory of Epidemiology of Major Diseases (Peking University), Ministry of Education, Beijing, China; 4 School of Nursing, Peking Union Medical College, Chinese Academy of Medical Sciences, 33 Ba Da Chu Road, Shijingshan District, Beijing, 100144 China

**Keywords:** Thromboembolism, Cancer, Sex disparities, Movement behaviors

## Abstract

**Background:**

Cancer patients face a markedly elevated risk of thromboembolism (TE), including both venous thromboembolism (VTE) and arterial thromboembolism (ATE), which contribute substantially to morbidity and mortality in this population.

**Objective:**

This study examined sex disparities in associations between sleep, sedentary behavior (SB), light physical activity (LPA), moderate-to-vigorous physical activity (MVPA), and TE risk, in cancer patients using data from the UK Biobank.

**Methods:**

A longitudinal cohort analysis of 6,765 cancer patients (2,774 men and 3,991 women) from the accelerometry subsample was conducted using Cox proportional hazards and isotemporal substitution models stratified by sex.

**Results:**

The incidence of VTE was 3.0% in men versus 2.2% in women, while ATE incidence was 5.0% versus 2.2%, respectively. Compared with high LPA, medium and low durations were associated with 2.75- and 2.88-fold higher VTE risk only in men. Reallocating 1 h per day from sleep or SB to LPA reduced VTE risk by 24% and 19% in men. Low MVPA was associated with 3.35- and 1.59-fold higher ATE risk in women and men, respectively. Reallocating 1 h per day from sleep, SB, or LPA to MVPA reduced ATE risk by 71%, 70%, and 66%, respectively, only in women.

**Conclusions:**

LPA was associated with a lower risk of VTE only in male cancer patients, whereas MVPA was linked to a lower risk of ATE in female patients, indicating sex-specific associations between movement behaviors and TE risk.

**Supplementary Information:**

The online version contains supplementary material available at 10.1186/s13293-026-00867-z.

## Highlights

1. This prospective cohort study identifies distinct sex-specific associations between movement behaviors and thromboembolism risk in cancer patients.
2. Higher light physical activity was associated with a lower risk of venous thromboembolism in men, particularly when replacing sleep or sedentary time.
3. Moderate-to-vigorous physical activity showed a stronger protective association with arterial thromboembolism in women than in men.
4. These findings highlight the importance of sex-tailored movement behavior strategies for thromboembolism prevention in cancer survivors.

**Supplementary Information:**

The online version contains supplementary material available at 10.1186/s13293-026-00867-z.

## Plain language summary

Patients with cancer have a substantially increased risk of thromboembolic events, including venous thromboembolism and arterial thromboembolism. These events can have serious consequences for health and survival. Physical activity and daily movement may help reduce this risk, but it remains unclear whether men and women with cancer benefit in the same way.In this study, we analyzed data from 6,765 adults with cancer enrolled in the UK Biobank. Patients wore a wrist-worn device that objectively measured time spent sleeping, sitting, performing light physical activities, and engaging in more intense physical activities. Participants were followed over time to identify the occurrence of venous and arterial thromboembolic events.We observed clear differences between men and women. In men with cancer, greater time spent in light physical activity was associated with a lower risk of venous thromboembolism. Replacing one hour per day of sleep or sitting with light activity was linked to a reduction in venous thromboembolism risk. This association was not observed in women.In contrast, moderate-to-vigorous physical activity played a more important role in women. Women with lower levels of moderate-to-vigorous-intensity activity had a higher risk of arterial thromboembolism. Replacing one hour per day of sleep, sitting, or light activity with more intense physical activity was associated with a reduction in arterial thromboembolism risk in women, while the effect was weaker in men.These findings suggest that movement-based prevention strategies for thromboembolism in cancer patients may benefit from a sex-specific approach.

**Supplementary Information:**

The online version contains supplementary material available at 10.1186/s13293-026-00867-z.

## Background

Thromboembolism (TE) is a vascular pathology characterized by thrombus formation and subsequent migration within the circulatory system, resulting in life-threatening complications and substantial global health burden [1, 2]. TE is one of the most prevalent cardiovascular diseases [3], particularly concerning in cancer patients [4–6], among whom cancer-associated TE represents the second leading cause of mortality [7–9]. Despite significant advancements over the past few decades, the incidence of TE in cancer patients remains alarmingly high [10, 11]. Cancer patients face an approximately 14-fold higher risk of venous thromboembolism (VTE) and a 6-fold higher risk of arterial thromboembolism (ATE) compared to individuals without cancer [12], with risk levels varying depending on cancer type and stage [13–15]. 

The health benefits of physical activity (PA) are well-established. For instance, patients with cancer are recommended to engage in at least 150 min of moderate-to-vigorous physical activity (MVPA) per week to reduce the risk of cancer-specific and all-cause mortality [16, 17]. Although the association between PA and TE risk has been previously explored [18–20], the role of movement behaviors in modulating cancer-related TE risk remains poorly understood. Clinical guidelines suggest that anticoagulant medications are effective in reducing the prevalence of TE [21–24], but they are also associated with an increased risk of hemorrhagic complications [25]. Given these risks, investigating alternative, non-pharmacological approaches, such as lifestyle intervention, for cancer-related TE prevention is needed.

Substantial sex disparities exist in both the epidemiology and clinical outcomes of cancer and cardiovascular disease [26–28]. These disparities likely arise from both biological differences and behavioral or social factors related to sex [26]. For example, females with colorectal cancer generally show better outcomes than males [27]. In cardiovascular disease, sex modifies the impact of both traditional and specific risk factors, highlighting the importance of considering sex in disease prevention and management [28]. Notably, sex-specific variations are also observed in the susceptibility to cardiotoxicity induced by anticancer therapies [29]. Furthermore, sex differences in cancer types and TE prevalence underscore the importance of gender disparities in the association between movement behaviors and cancer-related TE risk [30, 31]. 

Therefore, we aim to investigate sex disparities in the relationships between sleep, sedentary behavior (SB), light physical activity (LPA), MVPA, and TE risk in cancer patients, utilizing data from the UK Biobank. By leveraging objective accelerometer-based measurements, this study seeks to provide novel insights into the potential sex-specific effects of movement behaviors on TE risk.

## Methods

### Data source and study population

Data was obtained from the UK Biobank, a large prospective cohort study comprising 502 411 adults aged 40 to 69 years, and recruited from 22 assessment centers across the United Kingdom between 2006 and 2010. The study was approved by the North West Multi-Center Research Ethics Committee (Research Ethics Committee reference: 16/NW/0274) [32], with written informed consent obtained from all participants. Baseline data collection included anthropometric measurements, biospecimen sampling, and comprehensive lifestyle assessments. UK Biobank integrates data from national death registries, cancer registries, hospital inpatient records, and primary care records. All data were classified using standardized International Classification of Diseases-10th revision (ICD-10) and self-reported questionnaires to ensure the reliability and validity of cancer cases and research outcomes.

### PA measurement

PA was assessed using a triaxial accelerometer (Axivity AX3; Open Lab, Newcastle University) over a 7-day period. The device (dynamic range ± 8 g, sampling frequency 100 Hz) collected raw acceleration data, which were classified into 30-second epochs of sleep, SB, LPA, and MVPA using a validated machine learning model (Field ID: 40046–40049). Between February 2013 and December 2015, a subset of 236,519 participants was invited to wear wrist-worn accelerometers, of whom 103,579 provided valid data (Field ID: 90010), allowing for objective quantification of 24-hour movement behavior patterns [33, 34]. 

### Cancer ascertainment

As shown in Supplementary Table S1, cancer cases were identified based on the diagnosis of malignant neoplasms, as defined by ICD-10 codes C00-C97 (excluding non-melanoma skin cancers, coded as C44) [35, 36]. Data were sourced from national cancer registries linked to the UK Biobank, with diagnoses recorded before accelerometer measurements.

### Venous thromboembolism and arterial thromboembolism ascertainment

Incident VTE (including deep vein thrombosis or pulmonary embolism) [37] and ATE (including ischemic stroke or myocardial infarction) [38] events were identified based on ICD-10 codes and self-reported data, occurring after accelerometer measurements (see Supplementary Table S1). Participants were followed from accelerometer measurements until the occurrence of a VTE/ATE event, lost to follow-up, death, or December 31, 2021, whichever occurred first.

### Covariates

Covariates measured at baseline included age, race, education, body mass index (BMI), Townsend deprivation index, current smoking, current alcohol consumption, depressed mood, hypertension, diabetes, coronary heart disease, and stroke. Depressed mood was determined by self-reported feelings of depression or hopelessness occurring persistently (i.e., almost daily) or frequently (i.e., > 50% of the time) during the preceding two-week period. Hypertension was defined as either: (1) self-reported physician-diagnosed hypertension, (2) current use of antihypertensive medications, or (3) elevated blood pressure measurements at baseline (mean systolic blood pressure ≥ 140 mm Hg and/or diastolic blood pressure ≥ 90 mm Hg). Diabetes was diagnosed if participants met any of the following criteria: (1) self-reported physician diagnosis of diabetes (including type 1 or type 2 diabetes), (2) use of glucose-lowering agents, or (3) elevated glycemic parameters (glycated hemoglobin [HbA_1c_] ≥ 48 mmol/mol [≥ 6.5% of total hemoglobin]). History of coronary heart disease and stroke was ascertained through standardized self-reported questionnaires and confirmed by structured interviews conducted by trained medical staff during baseline assessments. Detailed description about the covariates was presented in Supplementary Table S2.

### Analytical sample

Participants with insufficient accelerometer wear time (*n* = 6938), inaccurate calibration (*n* = 1), implausibly high acceleration values (*n* = 3), > 1% clipped values before/after calibration (*n* = 13), missing data on BMI (*n* = 218) and the Townsend deprivation index (*n* = 107) were excluded. Additionally, cancer patients with a prior diagnosis of VTE (*n* = 311) or ATE (*n* = 231) before accelerometer measurements were excluded. The final analytical sample included 6765 participants (Supplementary Figure S1).

### Statistical analysis

Continuous variables are presented as means ± standard deviation (SD) or median with interquartile range (IQR), while categorical variables are expressed as frequencies and percentages. Student’s t-test and the Mann-Whitney U-test were used when appropriate, and the chi-square (χ²) test was used for categorical variables.

Sleep, SB, LPA, and MVPA were categorized into sex-specific tertiles based on the 33.3rd and 66.7th percentiles of their distributions (Supplementary Table S3). Multivariable-adjusted Cox proportional hazards regression models, stratified by sex, with follow-up time as the timescale, were used to test the association between sleep, SB, LPA, and MVPA and VTE/ATE risk in cancer patients. Additionally, isotemporal substitution Cox regression models, stratified by sex, were used to assess the impact of substituting one hour of daily SB, sleep, LPA, or MVPA with an equivalent duration of the other three variables on VTE and ATE risk in cancer patients. All movement behaviors and covariates were included in the models, except for the behavior being substituted. The coefficient for each behavior represents its effect on TE risk when replacing one hour per day of the omitted behavior with one hour per day of the specified behavior. The isotemporal substitution model is mathematically represented as follows:

$$\begin{aligned}&\text{TE risk }= \text{Intercept +b1}^*\mathrm{sleep} \\&+ \mathrm{b2}^*\text{SB + b3}^*\text{LPA + b4}^*\text{MVPA+ b} 5^*\mathrm{covariates}\end{aligned}$$

Models were adjusted for baseline variables, including age, race, educational level, BMI, Townsend deprivation index, smoking, alcohol use, depressed mood, hypertension, diabetes, coronary heart disease, and stroke. To test sex disparities in the regression coefficients derived from the Cox model, we applied the Z-test method introduced by Altman and Bland [39]. 

Additional sensitivity analyses were performed to evaluate the robustness of the findings. First, we examined the associations between overall physical activity (PA; LPA + MVPA) and VTE/ATE risk. Second, models were further adjusted for aspirin use to account for potential antithrombotic effects. Third, delayed-entry Cox regression models were conducted, with entry time defined as the interval between cancer diagnosis and accelerometer measurement, and exit time defined as the interval from cancer diagnosis to VTE/ATE occurrence, loss to follow-up, death, or December 31, 2021, whichever occurred first. Both Cox models and the isotemporal substitution models were fitted within this delayed-entry framework. Fourth, the delayed-entry Cox regression models were additionally stratified by survival duration (≤ 5 vs. > 5 years from cancer diagnosis to baseline) to assess the potential influence of survival time. Fifth, to account for competing risks, cause-specific Cox regression and isotemporal substitution analyses were performed, treating all-cause mortality as a competing event. Sixth, to evaluate the impact of exposure categorization, movement behaviors were classified using cut-points derived from the overall study population. The same cut-points were applied to both males and females. Finally, isotemporal substitution analyses were repeated using a smaller reallocation unit (30 min/day).

A two-sided *P*-value < 0.05 was considered statistically significant. All analyses were performed using SAS 9.4 (SAS Institute, Cary, NC) and R version 4.3.3 (R Foundation for Statistical Computing).

## Results

### Participant characteristics

A total of 2774 male cancer patients, with a median (IQR) age of 68.0 (64.0, 72.0) years, and 3991 female cancer patients, with a median (IQR) age of 66.0 (59.0, 70.0) years at the time of accelerometer measurements, were followed for a median of 7.1 years. During follow-up, 84 (3.0%) VTE events and 139 (5.0%) ATE events were recorded among male cancer patients, while 87 (2.2%) VTE events and 89 (2.2%) ATE events were recorded among female cancer patients. Compared to female cancer patients, male cancer patients were older, had a lower Townsend deprivation index, higher BMI, spent more time in SB and MVPA, less time in LPA, consumed more alcohol, and had higher prevalence rates of hypertension, diabetes, coronary heart disease, and stroke, and a lower rate of depressed mood (Table 1).

**Table 1:** Baseline Characteristics of Study Cancer Patients by Sex

Characteristics	Men (*n* = 2774)	Women (*n* = 3991)	*P*-Value
Age, median (IQR), y	68.0 (64.0, 72.0)	66.0 (59.0, 70.0)	< 0.001
Townsend index, median (IQR)	−2.7 (−3.9, −0.7)	−2.4 (−3.8, −0.3)	< 0.001
BMI, median (IQR)	26.7 (24.5, 29.2)	25.6 (23.1, 28.9)	< 0.001
SB, median (IQR), h	9.8 (8.6, 11.0)	9.2 (8.1, 10.3)	< 0.001
Sleep, median (IQR), h	8.8 (8.1, 9.7)	8.8 (8.2, 9.6)	0.364
LPA, median (IQR), h	4.4 (3.4, 5.5)	5.2 (4.2, 6.3)	< 0.001
MVPA, median (IQR), h	0.6 (0.3, 1.0)	0.4 (0.2, 0.8)	< 0.001
White race/ethnicity, n (%)	2706 (97.6)	3885 (97.3)	0.601
Higher educational level, n (%)	1639 (59.1)	2326 (58.3)	0.509
Current smoker, n (%)	186 (6.7)	230 (5.8)	0.113
Alcohol intake ≥once per week, n (%)	2288 (82.5)	2721 (68.2)	< 0.001
Depressed mood, n (%)	60 (2.2)	138 (3.5)	0.002
Hypertension, n (%)	1843 (66.4)	1935 (48.5)	< 0.001
Diabetes, n (%)	141 (5.1)	145 (3.6)	0.004
Coronary heart disease, n (%)	91 (3.3)	43 (1.1)	< 0.001
Stroke, n (%)	40 (1.4)	27 (0.7)	0.002

IQR, interquartile range; BMI, body Mass Index; SB, sedentary behavior; LPA, light physical activity; MVPA, moderate-to-vigorous physical activity

### Sex disparities in the associations between movement behaviors and VTE risk

**Fig. 1:**
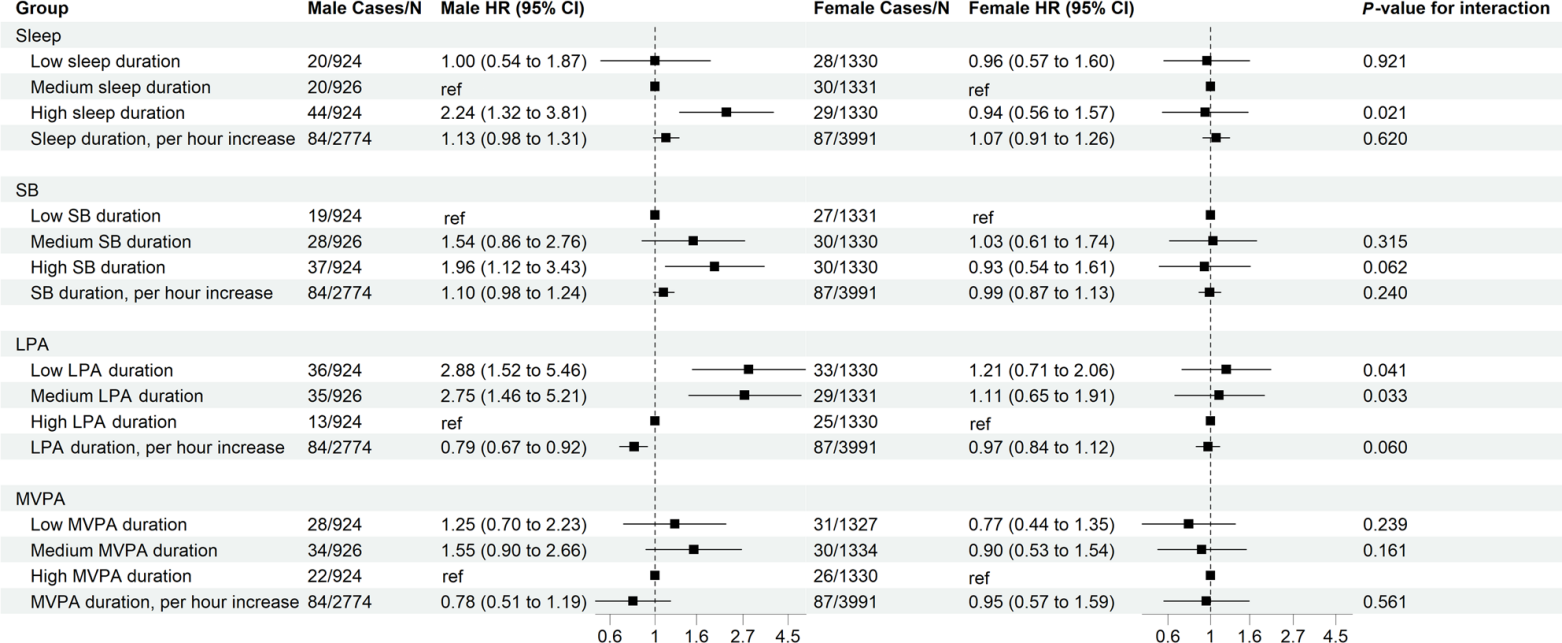
Associations Between SB, Sleep, LPA, and MVPA Duration and VTE Incidence Among Cancer Patients Stratified by Sex. Abbreviations: SB, sedentary behavior; LPA, light physical activity; MVPA, moderate-to-vigorous physical activity; VTE, venous thromboembolism.Adjusted covariates include age, race, education, body mass index, Townsend deprivation index, current smoking, current alcohol consumption, depressed mood, hypertension, diabetes, coronary heart disease, and stroke

As shown in Fig. 1, in the categorical analyses, medium (3.77 ≤ LPA duration ≤ 5.07 h/day) and low (LPA duration < 3.77 h/day) LPA durations were associated with a 2.75-fold (95% CI: 1.46–5.21) and 2.88-fold (95% CI: 1.52–5.46) higher risk of VTE, respectively, compared with high LPA duration (LPA duration > 5.07 h/day) among male cancer patients, while no significant associations were observed in females. Significant sex interactions were detected for both medium and low LPA durations (*P*-value for interaction = 0.033 and 0.041, respectively). Consistent results were observed when LPA was modeled as a continuous variable. Each 1-hour increase in LPA duration was associated with a 21% lower risk of incident VTE among male cancer patients (adjusted HR 0.79, 95% CI: 0.67–0.92), with no significant association in females. MVPA duration was not associated with VTE risk in either sex when modeled as a categorical or continuous variable.

**Table 2:** Hazard Ratios and 95% Confidence Intervals* for Incident VTE Estimated Using a Multivariable-Adjusted Isotemporal Substitution Cox Regression Model Among Cancer Patients Stratified by Sex

	Sleep	SB	LPA	MVPA
Men (*n* = 2774)				
Replace sleep with	Replaced	0.94 (0.82, 1.08)	0.76 (0.64, 0.90)	0.79 (0.52, 1.20)
Replace SB with	1.06 (0.92, 1.22)	Replaced	0.81 (0.69, 0.93)	0.83 (0.56, 1.24)
Replace LPA with	1.32 (1.11, 1.56)	1.24 (1.07, 1.44)	Replaced	1.03 (0.66, 1.61)
Replace MVPA with	1.27 (0.84, 1.94)	1.20 (0.81, 1.79)	0.97 (0.62, 1.51)	Replaced
Women (*n* = 3991)				
Replace sleep with	Replaced	0.94 (0.77, 1.16)	0.93 (0.75, 1.16)	0.92 (0.58, 1.45)
Replace SB with	1.06 (0.86, 1.30)	Replaced	0.98 (0.82, 1.18)	0.98 (0.64, 1.49)
Replace LPA with	1.08 (0.87, 1.34)	1.02 (0.85, 1.22)	Replaced	0.99 (0.63, 1.57)
Replace MVPA with	1.09 (0.70, 1.71)	1.03 (0.67, 1.57)	1.01 (0.64, 1.60)	Replaced

VTE, venous thromboembolism; SB, sedentary behavior; LPA, light physical activity; MVPA, moderate-to-vigorous physical activity

*Adjusted covariates include age, race, education, body mass index, Townsend deprivation index, current smoking, current alcohol consumption, depressed mood, hypertension, diabetes, coronary heart disease, and stroke

All substitutions represent reallocating 1 h per day from the column behavior to the row behavior

In the isotemporal substitution analyses (Table 2), reallocating 1 h/day from sleep (adjusted HR 0.76, 95% CI: 0.64–0.90) or sedentary behavior (SB) (adjusted HR 0.81, 95% CI: 0.69–0.93) to LPA was associated with a reduced VTE risk exclusively among male cancer patients.

### Sex disparities in the associations between movement behaviors and ATE risk

**Fig. 2:**
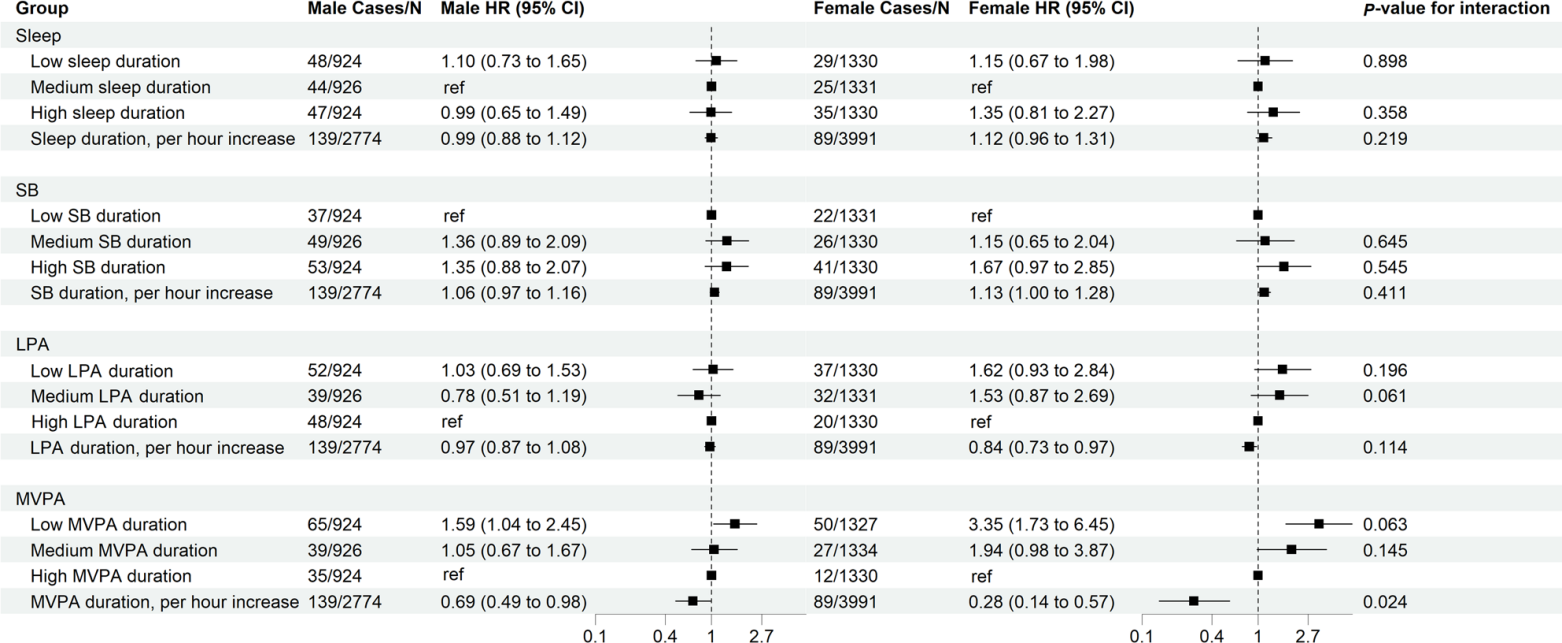
Associations Between SB, Sleep, LPA, and MVPA Duration and ATE Incidence Among Cancer Patients Stratified by Sex. Abbreviations: SB, sedentary behavior; LPA, light physical activity; MVPA, moderate-to-vigorous physical activity; ATE, arterial thromboembolism.Adjusted covariates include age, race, education, body mass index, Townsend deprivation index, current smoking, current alcohol consumption, depressed mood, hypertension, diabetes, coronary heart disease, and stroke

As illustrated in Fig. 2, in the categorical analyses, low MVPA duration (men: < 0.41 h/day; women: < 0.25 h/day) was associated with a 1.59-fold (95% CI: 1.04–2.45) and 3.35-fold (95% CI: 1.73–6.45) higher risk of ATE among male and female cancer patients, respectively, compared with high MVPA duration (men: > 0.87 h/day; women: > 0.62 h/day), with marginally significant sex difference (*P*-value for interaction = 0.063). When modeled continuously, each 1-hour increase in MVPA duration was associated with a 31% lower risk of ATE in males (adjusted HR 0.69, 95% CI: 0.49–0.98) and a 72% lower risk in females (adjusted HR 0.28, 95% CI: 0.14–0.57), with significant sex interaction (*P*-value for interaction = 0.024). LPA duration was not associated with ATE risk among males. In females, however, LPA duration modeled as a continuous variable was inversely associated with ATE risk (adjusted HR 0.84, 95% CI: 0.73–0.97).

In the isotemporal substitution analyses (Table 3), reallocating 1 h/day from sleep (adjusted HR 0.29, 95% CI: 0.14–0.60), SB (adjusted HR 0.30, 95% CI: 0.14–0.63), or LPA (adjusted HR 0.34, 95% CI: 0.16–0.72) to MVPA was associated with a reduced ATE risk exclusively among female cancer patients.

**Table 3:** Hazard Ratios and 95% Confidence Intervals* for Incident ATE Estimated Using a Multivariable-Adjusted Isotemporal Substitution Cox Regression Model Among Cancer Patients Stratified by Sex

	Sleep	SB	LPA	MVPA
Men (n=2774)				
Replace sleep with	Replaced	1.03 (0.90, 1.18)	1.00 (0.84, 1.18)	0.71 (0.48, 1.05)
Replace SB with	0.97 (0.85, 1.11)	Replaced	0.97 (0.86, 1.09)	0.69 (0.47, 1.01)
Replace LPA with	1.00 (0.84, 1.19)	1.03 (0.92, 1.16)	Replaced	0.71 (0.48, 1.07)
Replace MVPA with	1.41 (0.95, 2.08)	1.45 (0.99, 2.12)	1.40 (0.94, 2.10)	Replaced
Women (*n* = 3991)				
Replace sleep with	Replaced	0.97 (0.81, 1.16)	0.84 (0.69, 1.04)	0.29 (0.14, 0.60)
Replace SB with	1.03 (0.86, 1.23)	Replaced	0.87 (0.74, 1.01)	0.30 (0.14, 0.63)
Replace LPA with	1.19 (0.97, 1.46)	1.15 (0.99, 1.34)	Replaced	0.34 (0.16, 0.72)
Replace MVPA with	3.45 (1.68, 7.10)	3.36 (1.59, 7.10)	2.91 (1.39, 6.09)	Replaced

ATE, arterial thromboembolism; SB, sedentary behavior; LPA, light physical activity; MVPA, moderate-to-vigorous physical activity

*Adjusted covariates include age, race, education, body mass index, Townsend deprivation index, current smoking, current alcohol consumption, depressed mood, hypertension, diabetes, coronary heart disease, and stroke

All substitutions represent reallocating 1 h per day from the column behavior to the row behavior

### Sensitivity analyses

We conducted multiple sensitivity analyses to assess the robustness of our findings. First, Cox models examining total PA as a single exposure showed that only in male cancer patients, low and medium PA levels were associated with 2.9- and 1.9-fold higher VTE risk compared to high PA, whereas only in females, low PA was associated with a 2.0-fold higher ATE risk compared to high PA (Supplementary Figures S2-S3). Replacing sleep or SB with PA was associated with a lower VTE risk in males and a lower ATE risk in females (Supplementary Tables S4-S5). Second, further adjustment for aspirin use yielded results consistent with the primary analyses (Supplementary Figures S4-S5, Tables S6-S7). Third, delayed-entry models confirmed the main findings: in males, higher LPA was associated with lower VTE risk (HR 0.79, 95% CI 0.67–0.92), whereas in females, higher MVPA was associated with lower ATE risk (HR 0.28, 95% CI 0.13–0.56), with significant sex interactions (*P*-value for interaction = 0.047 and 0.027, respectively) (Supplementary Tables S8-S9). Replacing sleep or SB with LPA was associated with lower VTE risk in males, and replacing sleep, SB, or LPA with MVPA was associated with lower ATE risk in females (Supplementary Tables S10-S11). Fourth, stratified analyses by post-diagnosis survival time (≤ 5 vs. > 5 years) showed similar directional trends, although reduced sample size limited the statistical power to detect sex differences (Supplementary Tables S12-S15). Fifth, cause-specific Cox regression and isotemporal substitution analyses accounting for death as a competing event produced comparable results (Supplementary Tables S16-S17). In males, reallocating 1 h/day from sleep or sedentary behavior to LPA was associated with 22% and 18% lower VTE risk, respectively (Supplementary Table S18). In females, reallocating 1 h/day from sleep, sedentary behavior, or LPA to MVPA was associated with 70%, 69%, and 65% lower ATE risk, respectively (Supplementary Table S19). Sixth, analyses using population-wide tertiles for movement behaviors produced results consistent with the primary sex-specific tertile analyses: higher LPA was associated with lower VTE risk in males (HR 0.80, 95% CI 0.70–0.92), and higher MVPA was associated with lower ATE risk in females (HR 0.29, 95% CI 0.13–0.61) (Supplementary Tables S20-S21). Finally, isotemporal substitution analyses using a smaller reallocation unit of 30 min/day showed patterns similar to the 1-hour/day models: among males, reallocating 30 min/day from sleep or sedentary behavior to LPA was associated with 10% and 13% lower VTE risk, respectively; among females, reallocating 30 min/day from sleep, sedentary behavior, or LPA to MVPA was associated with 46%, 45%, and 41% lower ATE risk, respectively (Supplementary Tables S22-S23).

## Discussion

To our knowledge, few studies have examined sex disparities in the relationship between movement behaviors and TE risk in cancer survivors. In this prospective cohort study of 6,765 cancer patients from the UK Biobank, we observed significant sex-specific associations between movement behaviors and TE risk. Specifically, LPA was associated with a lower risk of VTE only in male patients, whereas MVPA was associated with a lower risk of ATE, particularly among female patients. These findings highlight the potential importance of sex-specific patterns of PA in the prevention of cancer-associated TE events.

To elucidate sex disparities in the association between movement behaviors and cancer-related TE risk, we used two analytical methods. First, we categorized sleep, SB, LPA, and MVPA into tertiles and compared their strengths of association with ATE and VTE across these groups. Our findings revealed that LPA reduced VTE risk only in males, whereas MVPA was more strongly associated with ATE risk reduction in females. Second, we applied an isotemporal substitution model, which yielded similar results. While existing literature has demonstrated that regular PA reduced VTE risk in the general population [40, 41], evidence regarding cancer-associated TE remains limited. Large-scale studies specifically addressing PA-TE associations in cancer populations are still lacking. Importantly, no previous research has systematically investigated potential gender disparities in this context. Our findings extend existing evidence by suggesting that LPA is a male-specific protective factor against VTE, whereas MVPA confers greater ATE protection in females. Importantly, isotemporal substitution models estimate theoretical reallocations of time between movement behaviors. These estimates should not be interpreted as prescriptive recommendations but rather as indicators of potential relationships, particularly given that substantial behavioral changes may not be feasible for all cancer patients.

The protective effect of LPA on VTE may be related to the following reasons. First, PA enhances blood flow, an adaptive response of the cardiovascular system to increased muscular demand, which may exert antithrombotic effects. Second, moderate exercise elevates fibrinolytic activity without significantly activating the coagulation system [42]. Third, prolonged intense exercise disrupts the balance between prothrombotic and fibrinolytic factors, resulting in a hypercoagulable state and impaired fibrinolysis [43]. Although similar physiological responses may apply to MVPA, several factors could explain its lack of association with VTE in our study. Cancer patients have limited MVPA due to fatigue, comorbidities, or functional impairment, reducing exposure variability and statistical power. High-intensity activity may also trigger transient procoagulant responses, partially offsetting antithrombotic benefits. In contrast, LPA is more sustainable and habitual, potentially promoting stable hemodynamic and fibrinolytic adaptations.

Our study confirmed that MVPA significantly lowers the risk of ATE, including myocardial infarction and stroke, consistent with prior epidemiological evidence [44]. The protective effects of MVPA may be mediated through several mechanisms, including (1) improved coagulation homeostasis, (2) reduced adiposity, and (3) amelioration of metabolic dysfunction. Moreover, MVPA may enhance cerebrovascular perfusion and endothelial function, thereby preserving cardiovascular and cerebrovascular integrity. Further mechanistic investigations suggest that MVPA modulates immune regulation and attenuates chronic low-grade inflammation, which could collectively contribute to its protective role against ATE [45]. Taken together, these findings underscore the importance of MVPA as a modifiable lifestyle factor for cancer-related ATE prevention.

However, the protective effect of LPA against VTE was observed only in male cancer patients, while MVPA was more protective against ATE in female cancer patients. These protective effects may be due to gender-specific differences in platelet function and varying risk of cancer-associated TE [46]. Furthermore, differences in cancer types and pathogenesis may be of importance. For instance, pancreatic, gastric, and primary brain cancers, which are more prevalent in males, are associated with a higher risk of VTE [47, 48]. Meanwhile, breast, endometrial, and ovarian cancers, which commonly occur in females, are strongly linked to estrogen-dependent pathways that may influence thrombosis risk through distinct hormonal mechanisms [49]. Additionally, variations in cancer treatments between genders may further contribute to these disparities. Given the substantial sex-based disparities in cancer type and the distinct pathophysiological mechanisms underlying cardiovascular events, further investigation is warranted to elucidate why there are sex disparities in the protective effects of movement behaviors against cancer-associated TE. Although anticoagulant therapy remains the cornerstone of TE prophylaxis [50], our findings suggest that movement behaviors, such as PA, may represent complementary factors associated with TE risk. Notably, these results should be considered hypothesis-generating and require confirmation in intervention studies, as behavioral feasibility and clinical applicability vary substantially across cancer populations.

A key strength of this study lies in the objective quantification of PA, SB, and sleep duration using wrist-worn accelerometers, which yield more accurate and reliable data than self-reported measures. Furthermore, exposures, outcomes, and covariates were assessed using standardized protocols with stringent quality control measures, thereby enhancing the validity of the association between movement behaviors and cancer-related TE risk. Our supplementary analysis generally confirms the shape of the associations in the main analysis, affirming the robustness of our results.

Several limitations should be acknowledged. First, participants in this study may not fully represent the general cancer population due to the age restriction (40–69 years) at UK Biobank recruitment. In addition, accelerometer data were available only for a subset of participants, with approximately 236,519 individuals invited and 103,579 providing valid measurements, corresponding to a response rate of 47.1% among those invited and approximately 20.6% of the overall cohort. This moderate participation may introduce selection bias and limit the generalizability of our findings. Second, the majority of participants were White and of higher socioeconomic status, limiting the generalizability of our findings. External validation in more diverse populations is warranted. Third, certain covariates, such as radiotherapy, chemotherapy, surgery, and anticoagulant use, were not fully accounted for, which may lead to confounding bias. Fourth, accelerometer measurements were performed only once, failing to capture long-term PA patterns. Reliance on a single-time measurement may introduce exposure misclassification, which would likely bias associations toward the null. Fifth, given the substantial heterogeneity in TE risk across cancer sites, stages, and treatment modalities, residual heterogeneity may have influenced the observed associations. Therefore, the reported estimates should be interpreted as average associations across heterogeneous cancer profiles rather than cancer-specific effects. Such heterogeneity is more likely to dilute true associations rather than generate spurious effects. Sixth, categorizing movement behaviors into sex-specific tertiles may complicate the interpretation of between-sex differences when absolute exposure levels differ. Nevertheless, continuous modeling and sensitivity analyses using common cut-points produced consistent results, supporting the robustness of our findings. Finally, except for age, covariates included in our analyses were obtained from the UK Biobank baseline assessment (2006–2010), while the analytical baseline was defined as the accelerometer assessment (2013–2015). Similar approaches have been widely used in prior studies, which have suggested that most covariates remain largely stable over time [51, 52]. 

## Conclusions

This longitudinal cohort study identified sex-specific associations between movement behaviors and TE risk among cancer patients. LPA was associated with a lower risk of VTE only in males, whereas MVPA was associated with a lower risk of ATE in females. Importantly, these sex-specific associations may inform targeted movement interventions to reduce thromboembolic risk, although caution is warranted given the observational design. Further confirmation in independent cohorts and interventional studies is warranted.

## Supplementary Information

Supplementary Material 1

## Data Availability

Data from the UK Biobank (https://www.ukbiobank.ac.uk/) are available to all researchers upon application.

## References

[1] Thrane PG, Olesen KKW, Thim T, Gyldenkerne C, Hansen MK, Stødkilde-Jørgensen N, et al. 10-Year Mortality After ST-Segment Elevation Myocardial Infarction Compared to the General Population. J Am Coll Cardiol. 2024;83:25:2615–25. 10.1016/j.jacc.2024.04.025. 38897670 10.1016/j.jacc.2024.04.025

[2] Keller K, Hobohm L, Ebner M, Kresoja K-P, Münzel T, Konstantinides SV, et al. Trends in thrombolytic treatment and outcomes of acute pulmonary embolism in Germany. Eur Heart J. 2020;41:4:522–9. 10.1093/eurheartj/ehz236. 31102407 10.1093/eurheartj/ehz236

[3] Danwang C, Temgoua MN, Agbor VN, Tankeu AT, Noubiap JJ. Epidemiology of venous thromboembolism in Africa: a systematic review. J Thromb Haemost. 2017;15:9:1770–81. 10.1111/jth.13769. 28796427 10.1111/jth.13769

[4] Frere C, Farge D, Schrag D, Prata PH, Connors JM. Direct oral anticoagulant versus low molecular weight heparin for the treatment of cancer-associated venous thromboembolism: 2022 updated systematic review and meta-analysis of randomized controlled trials. J Hematol Oncol. 2022;15:169. 10.1186/s13045-022-01289-1. 35598026 10.1186/s13045-022-01289-1 PMC9124390

[5] Koelwyn GJ, Newman AAC, Afonso MS, van Solingen C, Corr EM, Brown EJ, et al. Myocardial infarction accelerates breast cancer via innate immune reprogramming. Nat Med. 2020;26:9:1452–8. 10.1038/s41591-020-0964-7. 32661390 10.1038/s41591-020-0964-7 PMC7789095

[6] Costamagna G, Navi BB, Beyeler M, Hottinger AF, Alberio L, Michel P. Ischemic Stroke in Cancer: Mechanisms, Biomarkers, and Implications for Treatment. Semin Thromb Hemost. 2024;50:3342–59. 10.1055/s-0043-1771270. 10.1055/s-0043-1771270 37506734

[7] Donnellan E, Khorana AA. Cancer and Venous Thromboembolic Disease: A Review. Oncologist. 2017;22:2:199–207. 10.1634/theoncologist.2016-0214. 28174293 10.1634/theoncologist.2016-0214 PMC5330704

[8] Khorana AA, Francis CW, Culakova E, Kuderer NM, Lyman GH. Thromboembolism is a leading cause of death in cancer patients receiving outpatient chemotherapy. J Thromb Haemost. 2007;5:3:632–4. https://pubmed.ncbi.nlm.nih.gov/17319909. 17319909 10.1111/j.1538-7836.2007.02374.x

[9] Khorana AA. Venous thromboembolism and prognosis in cancer. Thromb Res. 2010;125(6):490–3. 10.1016/j.thromres.2009.12.023. 20097409 10.1016/j.thromres.2009.12.023 PMC2878879

[10] Hansen RS, Nybo M, Hvas A-M. Venous thromboembolism in pediatric cancer patients with central venous catheter-a systematic review and meta-analysis. Semin Thromb Hemost. 2021;47(8):920–30. 10.1055/s-0041-1729886. 34474495 10.1055/s-0041-1729886

[11] Icht O, Leader A, Batat E, Yosef L, Shochat T, Goldstein DA, et al. Arterial and venous thromboembolism in ALK-rearrangement-positive non-small cell lung cancer: a population-based cohort study. Oncologist. 2023;28(6):e391–6. 10.1093/oncolo/oyad061. 37014824 10.1093/oncolo/oyad061 PMC10243788

[12] Grilz E, Posch F, Nopp S, Königsbrügge O, Lang IM, Klimek P, et al. Relative risk of arterial and venous thromboembolism in persons with cancer vs. persons without cancer-a nationwide analysis. Eur Heart J. 2021;42(23):2299–307. 10.1093/eurheartj/ehab171. 33769475 10.1093/eurheartj/ehab171

[13] Petterson TM, Marks RS, Ashrani AA, Bailey KR, Heit JA. Risk of site-specific cancer in incident venous thromboembolism: a population-based study. Thromb Res. 2015;135(3):472–8. 10.1016/j.thromres.2014.12.013. 25547213 10.1016/j.thromres.2014.12.013 PMC4339484

[14] Black KA, Bowden S, Chu P, McClurg C, Pin S, Metcalfe A. Incidence of venous thromboembolism in patients with ovarian cancer receiving neoadjuvant chemotherapy: systematic review and meta-analysis. Int J Gynecol Cancer. 2024;34:6855–62. 10.1136/ijgc-2023-005166. 10.1136/ijgc-2023-005166 38431288

[15] Mulder FI, Horváth-Puhó E, van Es N, van Laarhoven HWM, Pedersen L, Moik F, et al. Venous thromboembolism in cancer patients: a population-based cohort study. Blood. 2021;137(14):1959–69. 10.1182/blood.2020007338. 33171494 10.1182/blood.2020007338

[16] Stout NL, Santa Mina D, Lyons KD, Robb K, Silver JK. A systematic review of rehabilitation and exercise recommendations in oncology guidelines. CA Cancer J Clin. 2021;71:2149–75. 10.3322/caac.21639. 10.3322/caac.21639 PMC7988887 33107982

[17] Rock CL, Thomson CA, Sullivan KR, Howe CL, Kushi LH, Caan BJ, et al. American Cancer Society nutrition and physical activity guideline for cancer survivors. CA Cancer J Clin. 2022;72(3):230–62. 10.3322/caac.21719. 35294043 10.3322/caac.21719

[18] Zhuang Z, Gao M, Yang R, Li N, Liu Z, Cao W, et al. Association of physical activity, sedentary behaviours and sleep duration with cardiovascular diseases and lipid profiles: a Mendelian randomization analysis. Lipids Health Dis. 2020;19(1):86. 10.1186/s12944-020-01257-z. 32384904 10.1186/s12944-020-01257-z PMC7206776

[19] Cowan LT, Tome J, Mallhi AK, Tarasenko YN, Palta P, Evenson KR, et al. Changes in physical activity and risk of ischemic stroke: the ARIC study. Int J Stroke. 2023;18(2):173–9. 10.1177/17474930221094221. 35361010 10.1177/17474930221094221 PMC9887651

[20] Ye R, Yang H, Li S, Ji C, Chen L, Zhao Y, et al. Accelerometer-measured intensity-specific physical activity, genetic predisposition, and the risk of venous thromboembolism: a cohort study. Eur J Prev Cardiol. 2025;32(1):65–74. 10.1093/eurjpc/zwae273. 39158115 10.1093/eurjpc/zwae273

[21] Streiff MB, Holmstrom B, Angelini D, Ashrani A, Elshoury A, Fanikos J, et al. Cancer-Associated Venous Thromboembolic Disease, Version 2.2021, NCCN Clinical Practice Guidelines in Oncology. J Natl Compr Canc Netw. 2021;19:101181–201. 10.6004/jnccn.2021.0047. 10.6004/jnccn.2021.0047 34666313

[22] Farge D, Frere C, Connors JM, Khorana AA, Kakkar A, Ay C, et al. 2022 international clinical practice guidelines for the treatment and prophylaxis of venous thromboembolism in patients with cancer, including patients with COVID-19. Lancet Oncol. 2022;23(7):e334–47. 10.1016/S1470-2045(22)00160-7. 35772465 10.1016/S1470-2045(22)00160-7 PMC9236567

[23] Falanga A, Ay C, Di Nisio M, Gerotziafas G, Jara-Palomares L, Langer F, et al. Venous thromboembolism in cancer patients: ESMO Clinical Practice Guideline. Ann Oncol. 2023;34(5):452–67. 10.1016/j.annonc.2022.12.014. 36638869 10.1016/j.annonc.2022.12.014

[24] Key NS, Khorana AA, Kuderer NM, Bohlke K, Lee AYY, Arcelus JI, et al. Venous Thromboembolism Prophylaxis and Treatment in Patients With Cancer: ASCO Guideline Update. J Clin Oncol. 2023;41(16):3063–71. 10.1200/JCO.23.00294. 37075273 10.1200/JCO.23.00294

[25] Ferrazzini E, Méan M, Stalder O, Limacher A, Rodondi N, Aujesky D. Incidence and clinical impact of bleeding events in older patients with acute venous thromboembolism. Blood Adv. 2023;7:2205–13. 10.1182/bloodadvances.2022007263. 10.1182/bloodadvances.2022007263 PMC9841039 35381071

[26] Siegfried JM. Sex and gender differences in lung cancer and chronic obstructive lung disease. Endocrinology. 2022. 10.1210/endocr/bqab254. 34927202 10.1210/endocr/bqab254

[27] Yang Y, Wang G, He J, Ren S, Wu F, Zhang J, et al. Gender differences in colorectal cancer survival: a meta-analysis. Int J Cancer. 2017;141(10):1942–9. 10.1002/ijc.30827. 28599355 10.1002/ijc.30827

[28] Rajendran A, Minhas AS, Kazzi B, Varma B, Choi E, Thakkar A, et al. Sex-specific differences in cardiovascular risk factors and implications for cardiovascular disease prevention in women. Atherosclerosis. 2023;384:117269. 10.1016/j.atherosclerosis.2023.117269. 37752027 10.1016/j.atherosclerosis.2023.117269 PMC10841060

[29] Wilcox NS, Rotz SJ, Mullen M, Song EJ, Ky Hamilton B, Moslehi J, et al. Sex-specific cardiovascular risks of cancer and its therapies. Circ Res. 2022;130(4):632–51. 10.1161/CIRCRESAHA.121.319901. 35175846 10.1161/CIRCRESAHA.121.319901 PMC8915444

[30] Yoshikawa T, Sano T, Terashima M, Yamaguchi K, Bando E, Kawabata R, et al. Incidence and risk factors for venous thromboembolism in the Cancer-VTE Registry stomach cancer subcohort. Gastric Cancer. 2023;26(4):493–503. 10.1007/s10120-023-01378-1. 37004667 10.1007/s10120-023-01378-1 PMC10284943

[31] Giustozzi M, Valerio L, Agnelli G, Becattini C, Fronk E-M, Klok FA, et al. Sex-specific differences in the presentation, clinical course, and quality of life of patients with acute venous thromboembolism according to baseline risk factors. Insights from the PREFER in VTE. Eur J Intern Med. 2021;88:43–51. 10.1016/j.ejim.2021.03.014. 33810940 10.1016/j.ejim.2021.03.014

[32] Sudlow C, Gallacher J, Allen N, Beral V, Burton P, Danesh J, et al. UK biobank: an open access resource for identifying the causes of a wide range of complex diseases of middle and old age. PLoS Med. 2015;12:3e1001779. 10.1371/journal.pmed.1001779. 10.1371/journal.pmed.1001779 PMC4380465 25826379

[33] Shreves AH, Small SR, Travis RC, Matthews CE, Doherty A. Dose-response of accelerometer-measured physical activity, step count, and cancer risk in the UK Biobank: a prospective cohort analysis. Lancet (London England). 2023;402(Suppl 1):S83. 10.1016/S0140-6736(23)02147-5. 37997129 10.1016/S0140-6736(23)02147-5

[34] Zhang X, Liu Y-M, Lei F, Huang X, Liu W, Sun T, et al. Association between questionnaire-based and accelerometer-based physical activity and the incidence of chronic kidney disease using data from UK Biobank: a prospective cohort study. EClinicalMedicine. 2023;66:102323. 10.1016/j.eclinm.2023.102323. 38024479 10.1016/j.eclinm.2023.102323 PMC10679485

[35] Sanchez-Lastra MA, Strain T, Ding D, Dalene KE, Del Pozo Cruz B, Ekelund U, et al. Associations of adiposity and device-measured physical activity with cancer incidence: UK Biobank prospective cohort study. J Sport Health Sci. 2024;14:101018. 10.1016/j.jshs.2024.101018. 39675506 10.1016/j.jshs.2024.101018 PMC11981806

[36] Kantor ED, O’Connell K, Du M, Mendelsohn RB, Liang PS, Braunstein LZ. Ranitidine Use and Cancer Risk: Results From UK Biobank. Gastroenterology. 2021;160:5. 10.1053/j.gastro.2020.12.037. 10.1053/j.gastro.2020.12.037 PMC8035224 33385434

[37] Khan F, Tritschler T, Kahn SR, Rodger MA. Venous thromboembolism. Lancet (London England). 2021;398:10294:64–77. 10.1016/S0140-6736(20)32658-1. 33984268 10.1016/S0140-6736(20)32658-1

[38] Gervaso L, Dave H, Khorana AA. Venous and Arterial Thromboembolism in Patients With Cancer: JACC: CardioOncology State-of-the-Art Review. JACC CardioOncol. 2021;3:2. 10.1016/j.jaccao.2021.03.001. 10.1016/j.jaccao.2021.03.001 PMC8352228 34396323

[39] Altman DG, Bland JM. Interaction revisited: the difference between two estimates. BMJ (Clinical Res ed). 2003;326(7382):219. https://pubmed.ncbi.nlm.nih.gov/12543843. 10.1136/bmj.326.7382.219 PMC1125071 12543843

[40] Kunutsor SK, Mäkikallio TH, Seidu S, de Araújo CGS, Dey RS, Blom AW, et al. Physical activity and risk of venous thromboembolism: systematic review and meta-analysis of prospective cohort studies. Eur J Epidemiol. 2020;35:5431–42. 10.1007/s10654-019-00579-2. 10.1007/s10654-019-00579-2 PMC7250794 31728878

[41] Chiu TS, Pankow JS, Cushman M, Windham BG, Matsushita K, Mok Y, et al. Frailty and risk of venous thromboembolism in older adults: the Atherosclerosis Risk in Communities Study. J Thromb Haemost. 2025. 10.1016/j.jtha.2025.01.006. 39894445 10.1016/j.jtha.2025.01.006 PMC12043403

[42] Nagelkirk PR, Soave K, Altherr C, Del Pozzi A. Regular resistance training enhances fibrinolytic potential but does not affect coagulation. Med Sci Sports Exerc. 2021;53(11):2318–23. 10.1249/MSS.0000000000002724. 34115732 10.1249/MSS.0000000000002724

[43] Kicken CH, Miszta A, Kelchtermans H, De Laat B. Hemostasis during extreme exertion. Semin Thromb Hemost. 2018;44(7):640–50. 10.1055/s-0038-1639502. 29727892 10.1055/s-0038-1639502

[44] Peter-Marske KM, Evenson KR, Moore CC, Cuthbertson CC, Howard AG, Shiroma EJ, et al. Association of accelerometer-measured physical activity and sedentary behavior with incident cardiovascular disease, myocardial infarction, and ischemic stroke: the Women’s Health Study. J Am Heart Assoc. 2023;12(7):e028180. 10.1161/JAHA.122.028180. 36974744 10.1161/JAHA.122.028180 PMC10122899

[45] Wang Y, Han Q, Han X, Dong Y, Mao M, Wang C, et al. Objectively-measured movement behaviors, systemic low-grade inflammation, and plasma neurofilament light chain in older adults: a population-based study. Immun Ageing. 2023;20(1):36. 10.1186/s12979-023-00363-7. 37491244 10.1186/s12979-023-00363-7 PMC10367375

[46] Huskens D, Roest M, Remijn JA, Konings J, Kremers RMW, Bloemen S, et al. Strenuous exercise induces a hyperreactive rebalanced haemostatic state that is more pronounced in men. Thromb Haemost. 2016;115(6):1109–19. 10.1160/TH15-10-0821. 26864794 10.1160/TH15-10-0821

[47] Fan H, Yu Y, Nan H, Hoyt M, Reger MK, Prizment A, et al. Associations between intake of calcium, magnesium and phosphorus and risk of pancreatic cancer: a population-based, case-control study in Minnesota. Br J Nutr. 2021;126(10):1549–57. 10.1017/S0007114521000283. 33494844 10.1017/S0007114521000283

[48] Lee JK, Merchant SA, Schneider JL, Jensen CD, Fireman BH, Quesenberry CP, et al. Proton pump inhibitor use and risk of gastric, colorectal, liver, and pancreatic cancers in a community-based population. Am J Gastroenterol. 2020;115(5):706–15. 10.14309/ajg.0000000000000591. 32205645 10.14309/ajg.0000000000000591

[49] Muñoz AJ, de Toro M, Ortega L, López C, Gutiérrez A, Juliao DS, et al. Venous thromboembolism incidence in cancer patients with germline BRCA mutations. Clin Transl Oncol. 2022;24(1):154–8. 10.1007/s12094-021-02678-7. 34374030 10.1007/s12094-021-02678-7

[50] Wei Q, Wei Z-Q, Jing C-Q, Li Y-X, Zhou D-B, Lin M-B, et al. Incidence, prevention, risk factors, and prediction of venous thromboembolism in Chinese patients after colorectal cancer surgery: a prospective, multicenter cohort study. Int J Surg. 2023;109(10):3003–12. 10.1097/JS9.0000000000000553. 37338597 10.1097/JS9.0000000000000553 PMC10583908

[51] Kim Y, Jang H, Wang M, Shi Q, Strain T, Sharp SJ, et al. Replacing device-measured sedentary time with physical activity is associated with lower risk of coronary heart disease regardless of genetic risk. J Intern Med. 2023;295(1):38–50. 10.1111/joim.13715. 37614046 10.1111/joim.13715 PMC10953003

[52] Strain T, Wijndaele K, Dempsey PC, Sharp SJ, Pearce M, Jeon J, et al. Wearable-device-measured physical activity and future health risk. Nat Med. 2020;26(9):1385–91. 10.1038/s41591-020-1012-3. 32807930 10.1038/s41591-020-1012-3 PMC7116559

