# Supplementary material for: Sex disparities in the associations between accelerometer-measured movement behaviors and subsequent thromboembolism risk in cancer patients
Source: Biol Sex Differ. 2026 Mar 2;17:66. doi: 10.1186/s13293-026-00867-z (PMC13059444; doi:10.1186/s13293-026-00867-z)
Supplement: Supplementary file 1 — Supplementary Material 1 [file 13293_2026_867_MOESM1_ESM.docx]

**Supplementary Material**

Figure S1. Flowchart of Participant Selection

Table S1. Code Lists for VTE, ATE, and Cancer

Table S2. Definition and Assessment of Covariates

Table S3. Three Equivalent Values of Sleep, SB, LPA, and MVPA in Male and Female Cancer Patients

Figure S2. Associations Between PA Duration and VTE Incidence Among Cancer Patients Stratified by Sex

Table S4. Hazard Ratios and 95% Confidence Intervals* for Incident VTE Estimated From a Multivariable-Adjusted Isotemporal Substitution Cox Regression Model Using Total Physical Activity Among Cancer Patients Stratified by Sex

Figure S3. Associations Between PA Duration and ATE Incidence Among Cancer Patients Stratified by Sex

Table S5. Hazard Ratios and 95% Confidence Intervals* for Incident ATE Estimated From a Multivariable-Adjusted Isotemporal Substitution Cox Regression Model Using Total Physical Activity Among Cancer Patients Stratified by Sex

Figure S4. Associations Between SB, Sleep, LPA, and MVPA Duration and VTE Incidence Among Cancer Patients Stratified by Sex Further Adjusted for Aspirin

Table S6. Hazard Ratios and 95% Confidence Intervals* for Incident VTE Estimated Using a Multivariable-Adjusted Isotemporal Substitution Cox Regression Model Among Cancer Patients Stratified by Sex (Further Adjusted for Aspirin)

Figure S5. Associations Between SB, Sleep, LPA, and MVPA Duration and ATE Incidence Among Cancer Patients Stratified by Sex Further Adjusted for Aspirin

Table S7. Hazard Ratios and 95% Confidence Intervals* for Incident ATE Estimated Using a Multivariable-Adjusted Isotemporal Substitution Cox Regression Model Among Cancer Patients Stratified by Sex (Further Adjusted for Aspirin)

Table S8. Association between Movement Behaviors and VTE Incidence among Cancer Patients Stratified by Sex: Delayed-Entry Cox Models

Table S9. Association between Movement Behaviors and ATE Incidence among Cancer Patients Stratified by Sex: Delayed-Entry Cox Models

Table S10 Hazard Ratios* for Incident VTE Estimated Using Multivariable-Adjusted Delayed-Entry Isotemporal Substitution Cox Regression Models Among Cancer Patients Stratified by Sex

Table S11. Hazard Ratios* for Incident ATE Estimated Using Multivariable-Adjusted Delayed-Entry Isotemporal Substitution Cox Regression Models Among Cancer Patients Stratified by Sex

Table S12. Association between Movement Behaviors and VTE Incidence among Cancer Patients Stratified by Sex and Cancer Diagnosis to Baseline Duration (≤5 years): Delayed-Entry Cox Models

Table S13. Association between Movement Behaviors and ATE Incidence among Cancer Patients Stratified by Sex and Cancer Diagnosis to Baseline Duration (≤5 years): Delayed-Entry Cox Models

Table S14. Association between Movement Behaviors and VTE Incidence among Cancer Patients Stratified by Sex and Cancer Diagnosis to Baseline Duration (>5 years): Delayed-Entry Cox Models

Table S15. Association between Movement Behaviors and ATE Incidence among Cancer Patients Stratified by Sex and Cancer Diagnosis to Baseline Duration (>5 years): Delayed-Entry Cox Models

Table S16. Association between SB, Sleep, LPA, and MVPA Duration and Incident VTE Among Cancer Patients Stratified by Sex: Competing-Risk Models

Table S17. Association between SB, Sleep, LPA, and MVPA Duration and Incident ATE Among Cancer Patients Stratified by Sex: Competing-Risk Models

Table S18 Hazard Ratios* for Incident VTE Estimated Using Multivariable-Adjusted Isotemporal Substitution Competing-Risk Models Among Cancer Patients Stratified by Sex

Table S19. Hazard Ratios* for Incident ATE Estimated Using Multivariable-Adjusted Isotemporal Substitution Competing-Risk Models Among Cancer Patients Stratified by Sex

Table S20. Association between SB, Sleep, LPA, and MVPA Duration and VTE Incidence Among Cancer Patients Stratified by Sex Using Tertiles Derived from the Overall Cancer Sample

Table S21. Association between SB, Sleep, LPA, and MVPA Duration and ATE Incidence Among Cancer Patients Stratified by Sex Using Tertiles Derived from the Overall Cancer Sample

Table S22 Hazard Ratios* for Incident VTE Estimated Using a Multivariable-Adjusted Isotemporal Substitution Cox Regression Model (30 min/day Reallocation) Among Cancer Patients Stratified by Sex

Table S23. Hazard Ratios* for Incident ATE Estimated Using a Multivariable-Adjusted Isotemporal Substitution Cox Regression Model (30 min/day Reallocation) Among Cancer Patients Stratified by Sex

Figure S1. Flowchart of Participant Selection


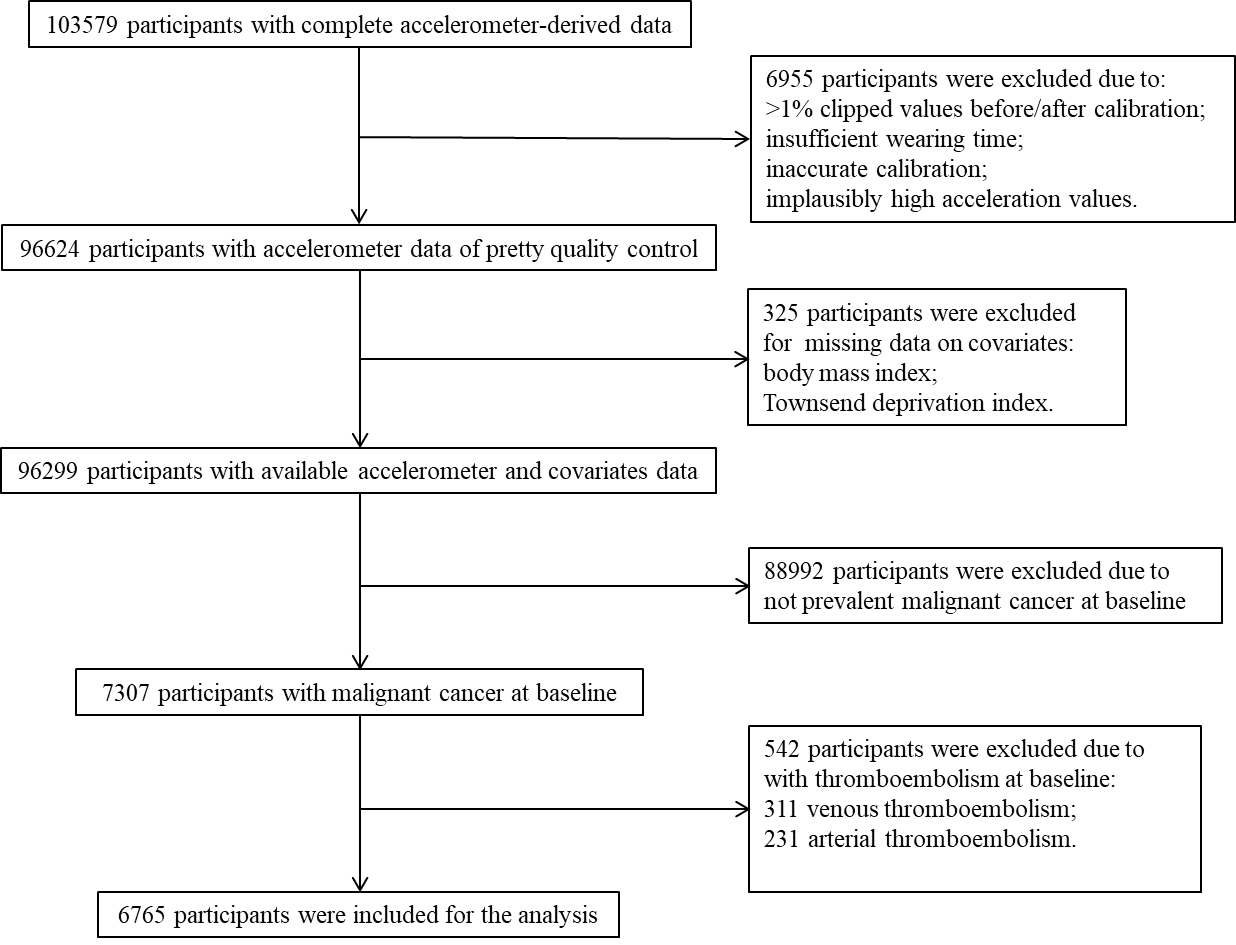


Table S1. Code Lists for VTE, ATE, and Cancer

|  | Code description | Category |
| --- | --- | --- |
| ICD 10 Code |  | VTE |
| I80 | Phlebitis and thrombophlebitis | DVT |
| I80.1 | Phlebitis and thrombophlebitis of femoral vein | DVT |
| I80.2 | Phlebitis and thrombophlebitis of other deep vessels of lower extremities | DVT |
| I80.3 | Phlebitis and thrombophlebitis of lower extremities, unspecified | DVT |
| I80.8 | Phlebitis and thrombophlebitis of other sites | DVT |
| I80.9 | Phlebitis and thrombophlebitis of unspecified site | DVT |
| I81 | Portal vein thrombosis | DVT |
| I82.0 | Budd-Chiari syndrome | DVT |
| I82.2 | Embolism and thrombosis of vena cava | DVT |
| I82.3 | Embolism and thrombosis of renal vein | DVT |
| I82.8 | Embolism and thrombosis of other specified veins | DVT |
| I82.9 | Embolism and thrombosis of unspecified vein | DVT |
| I67.6 | Nonpyogenic thrombosis of intracranial venous system | DVT |
| O22.3 | Deep phlebothrombosis in pregnancy | DVT |
| O22.5 | Cerebral venous thrombosis in pregnancy | DVT |
| O87.1 | Deep phlebothrombosis in the puerperium | DVT |
| I26.0 | Pulmonary embolism with mention of acute cor pulmonale | PE |
| I26.9 | Pulmonary embolism without mention of acute cor pulmonale | PE |
|  |  | Cancer |
| C00-C14 | Malignant neoplasms of lip, oral cavity and pharynx | Cancer |
| C15-C26 | Malignant neoplasms of digestive organs | Cancer |
| C30-C39 | Malignant neoplasms of respiratory and intrathoracic organs | Cancer |
| C40-C41 | Malignant neoplasms of bone and articular cartilage | Cancer |
| C43-C43 | Malignant melanoma of skin | Cancer |
| C45-C49 | Malignant neoplasms of mesothelial and soft tissue | Cancer |
| C50-C50 | Malignant neoplasm of breast | Cancer |
| C51-C58 | Malignant neoplasms of female genital organs | Cancer |
| C60-C63 | Malignant neoplasms of male genital organs | Cancer |
| C64-C68 | Malignant neoplasms of urinary tract | Cancer |
| C69-C72 | Malignant neoplasms of eye, brain and other parts of central nervous system | Cancer |
| C73-C75 | Malignant neoplasms of thyroid and other endocrine glands | Cancer |
| C76-C80 | Malignant neoplasms of ill-defined, secondary and unspecified sites | Cancer |
| C81-C96 | Malignant neoplasms, stated or presumed to be primary, of lymphoid, haematopoietic and related tissue | Cancer |
| C97-C97 | Malignant neoplasms of independent (primary) multiple sites | Cancer |
|  |  | ATE |
| I63 | Cerebral infarction | IS |
| I63.0 | Cerebral infarction due to thrombosis of precerebral arteries | IS |
| I63.1 | Cerebral infarction due to embolism of precerebral arteries | IS |
| I63.2 | Cerebral infarction due to unspecified occlusion or stenosis of precerebral arteries | IS |
| I63.3 | Cerebral infarction due to thrombosis of cerebral arteries | IS |
| I63.4 | Cerebral infarction due to embolism of cerebral arteries | IS |
| I63.5 | Cerebral infarction due to unspecified occlusion or stenosis of cerebral arteries | IS |
| I63.6 | Cerebral infarction due to cerebral venous thrombosis, nonpyogenic | IS |
| I63.8 | Other cerebral infarction | IS |
| I63.9 | Cerebral infarction, unspecified | IS |
| I64.X | Stroke, not specified as haemorrhage or infarction | IS |
| I21 | Acute myocardial infarction | MI |
| I21.0 | Acute transmural myocardial infarction of anterior wall | STEMI |
| I21.1 | Acute transmural myocardial infarction of inferior wall | STEMI |
| I21.2 | Acute transmural myocardial infarction of other sites | STEMI |
| I21.3 | Acute transmural myocardial infarction of unspecified site | STEMI |
| I21.4 | Acute subendocardial myocardial infarction | NSTEMI |
| I21.9 | Acute myocardial infarction, unspecified | NSTEMI |
| I22 | Subsequent myocardial infarction | MI |
| I22.0 | Subsequent myocardial infarction of anterior wall | STEMI |
| I22.1 | Subsequent myocardial infarction of inferior wall | STEMI |
| I22.8 | Subsequent myocardial infarction of other sites | NSTEMI |
| I22.9 | Subsequent myocardial infarction of unspecified site | MI |
| I23 | Certain current complications following acute myocardial infarction | MI |
| I23.0 | Haemopericardium as current complication following acute myocardial infarction | MI |
| I23.1 | Atrial septal defect as current complication following acute myocardial infarction | MI |
| I23.2 | Ventricular septal defect as current complication following acute myocardial infarction | MI |
| I23.3 | Rupture of cardiac wall without haemopericardium as current complication following acute myocardial infarction | MI |
| I23.4 | Rupture of chordae tendineae as current complication following acute myocardial infarction | MI |
| I23.5 | Rupture of papillary muscle as current complication following acute myocardial infarction | MI |
| I23.6 | Thrombosis of atrium, auricular appendage, and ventricle as current complications following acute myocardial infarction | MI |
| I23.8 | Other current complications following acute myocardial infarction | MI |
| I24.1 | Dressler syndrome | MI |
| I25.2 | Old myocardial infarction | MI |
| Self-report |  | VTE |
| 1094 | Deep venous thrombosis | DVT |
| 1093 | Pulmonary embolism | PE |

Abbreviations: VTE, venous thromboembolism; DVT, deep vein thrombosis; PE, pulmonary embolism; IS, ischaemic stroke; MI, myocardial infarction; STEMI, ST-segment elevation myocardial infarction; NSTEMI, non-ST-segment elevation myocardial infarction.

Table S2. Definition and Assessment of Covariates

| Covariates | Definition | Assessment | UK biobank Data-Field ID |
| --- | --- | --- | --- |
| Age | Age in years | Difference between date attended baseline assessment and date of birth recorded by NHS | 21003 |
| Sex | Men, Women | NHS derived and/or touchscreen questionnaire | 31 |
| Race | White, Nonwhite (Mixed, Asian, Black, Chinese, Other) | Touchscreen questionnaire: “What is your ethnic group?” | 21000 |
| Education | Higher education (college or university degree, other professional qualifications), other than higher education | Touchscreen questionnaire: “Which of the following qualifications do you have?” | 6138 |
| Body mass index (kg/m^2^) | Continuous | Physical examination: body mass index, calculated from height and weight measured during the initial Assessment Centre visit. | 21001 |
| Townsend deprivation index | Continuous | Townsend deprivation index calculated before participant joining UK Biobank. According to the preceding national census output areas. Each participant is assigned a score based on the output area in which their postcode is located. | 22189 |
| Current smoking | Yes, No | Touchscreen questionnaire: “Do you smoke tobacco now?” and “In the past, how often have you smoked tobacco?” | 20116 |
| Current alcohol consumption | At least once per week, less than once per week | Touchscreen questionnaire: “About how often do you drink alcohol?” | 1558 |
| Depressed mood | Yes (nearly every day or more than half the days), No (not at all or several days) | Touchscreen questionnaire: “Over the past two weeks, how often have you felt down, depressed or hopeless?” | 2050 |
| Hypertension | Yes, No | Touchscreen questionnaire and verbal interview: self-reported hypertension or anti-hypertensive medication use; Average SBP/DBP ≥ 140/90 mmHg at baseline | 6150, 20002, 6177, 4079, 4080, 93, 94 |
| Diabetes | Yes, No | Touchscreen questionnaire and verbal interview: self-reported diabetes (diabetes, type 1 diabetes or type 2 diabetes) or medication use for lowering blood glucose; Plasma HbA1c ≥ 48 mmol/mol (6.5%) | 2443, 20002, 6153, 6177, 30750, 20003 |
| Coronary heart disease | Yes, No | Touchscreen questionnaire and verbal interview: angina, heart attack/myocardial infarction | 6150, 20002 |
| Stroke | Yes, No | Touchscreen questionnaire and verbal interview: self-reported previous stroke or transient ischemic attack (TIA) | 6150, 20002 |
| Aspirin | Yes, No | Verbal interview: self-reported Aspirin use | 20003 |

Table S3. Three Equivalent Values of Sleep, SB, LPA, and MVPA in Cancer Patients

| Variable, hour/day | Men | Women | Overall |
| --- | --- | --- | --- |
| Sleep |  |  |  |
| Low sleep duration | <8.33 | <8.39 | <8.36 |
| Medium sleep duration | 8.33-9.34 | 8.39-9.32 | 8.36-9.32 |
| High sleep duration | >9.34 | >9.32 | >9.32 |
| SB |  |  |  |
| Low SB duration | <9.02 | <8.50 | <8.70 |
| Medium SB duration | 9.02-10.54 | 8.50-9.92 | 8.70-10.20 |
| High SB duration | >10.54 | >9.92 | >10.20 |
| LPA |  |  |  |
| Low LPA duration | <3.77 | <4.52 | <4.19 |
| Medium LPA duration | 3.77-5.07 | 4.52-5.89 | 4.19-5.57 |
| High LPA duration | >5.07 | >5.89 | >5.57 |
| MVPA |  |  |  |
| Low MVPA duration | <0.41 | <0.25 | <0.30 |
| Medium MVPA duration | 0.41-0.87 | 0.25-0.62 | 0.30-0.72 |
| High MVPA duration | >0.87 | >0.62 | >0.72 |

Abbreviations: SB, sedentary behavior; LPA, light physical activity; MVPA, moderate-to-vigorous physical activity; PA, physical activity

Figure S2. Associations Between PA Duration and VTE Incidence Among Cancer Patients Stratified by Sex


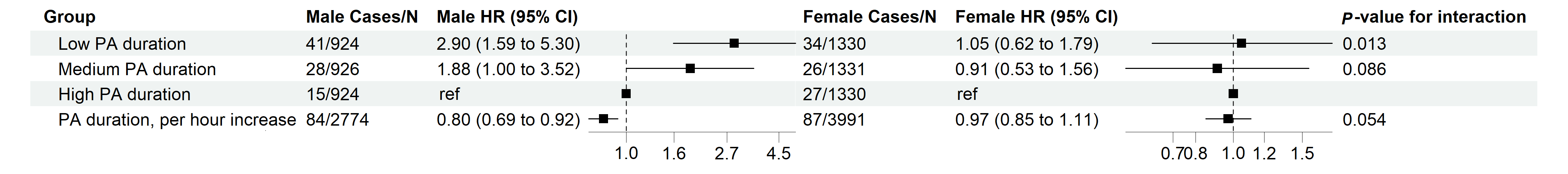
Abbreviations: PA, physical activity; VTE, venous thromboembolism.

Adjusted covariates include age, race, education, body mass index, Townsend deprivation index, current smoking, current alcohol consumption, depressed mood, hypertension, diabetes, coronary heart disease, and stroke.

Table S4. Hazard Ratios and 95% Confidence Intervals* for Incident VTE Estimated From a Multivariable-Adjusted Isotemporal Substitution Cox Regression Model Using Total Physical Activity Among Cancer Patients Stratified by Sex

|  | Sleep | SB | PA |
| --- | --- | --- | --- |
| Men (n=2774) |  | | |
| Replace sleep with | Replaced | 0.94 (0.82, 1.08) | 0.76 (0.65, 0.90) |
| Replace SB with | 1.06 (0.92, 1.22) | Replaced | 0.81 (0.71, 0.92) |
| Replace PA with | 1.31 (1.11, 1.54) | 1.24 (1.08, 1.41) | Replaced |
| Women (n=3991) |  | | |
| Replace sleep with | Replaced | 0.94 (0.77, 1.16) | 0.93 (0.75, 1.15) |
| Replace SB with | 1.06 (0.86, 1.30) | Replaced | 0.98 (0.83, 1.16) |
| Replace PA with | 1.08 (0.87, 1.33) | 1.02 (0.86, 1.21) | Replaced |

Abbreviations: VTE, venous thromboembolism; SB, sedentary behavior; PA, physical activity.

*Adjusted covariates include age, race, education, body mass index, Townsend deprivation index, current smoking, current alcohol consumption, depressed mood, hypertension, diabetes, coronary heart disease, and stroke.

All substitutions represent reallocating 1 hour per day from the column behavior to the row behavior.

Figure S3. Associations Between PA Duration and ATE Incidence Among Cancer Patients Stratified by Sex


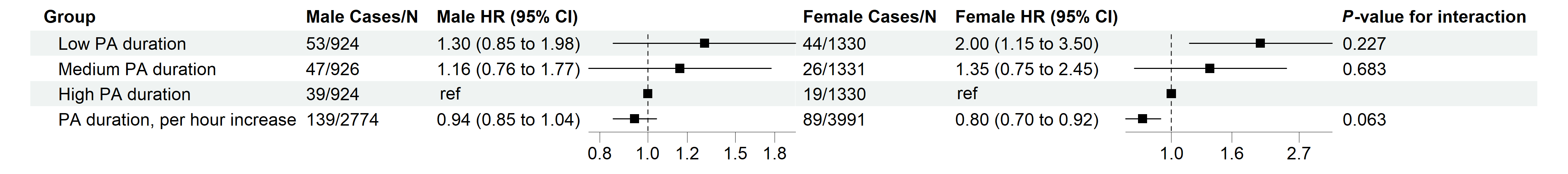
Abbreviations: PA, physical activity; ATE, arterial thromboembolism.

Adjusted covariates include age, race, education, body mass index, Townsend deprivation index, current smoking, current alcohol consumption, depressed mood, hypertension, diabetes, coronary heart disease, and stroke.

Table S5. Hazard Ratios and 95% Confidence Intervals* for Incident ATE Estimated From a Multivariable-Adjusted Isotemporal Substitution Cox Regression Model Using Total Physical Activity Among Cancer Patients Stratified by Sex

|  | Sleep | SB | PA |
| --- | --- | --- | --- |
| Men (n=2774) |  | | |
| Replace sleep with | Replaced | 1.03 (0.90, 1.19) | 0.96 (0.81, 1.14) |
| Replace SB with | 0.97 (0.84, 1.11) | Replaced | 0.93 (0.83, 1.05) |
| Replace PA with | 1.04 (0.88, 1.23) | 1.07 (0.96, 1.20) | Replaced |
| Women (n=3991) |  | | |
| Replace sleep with | Replaced | 0.97 (0.81, 1.16) | 0.78 (0.64, 0.96) |
| Replace SB with | 1.03 (0.86, 1.23) | Replaced | 0.81 (0.69, 0.95) |
| Replace PA with | 1.28 (1.04, 1.57) | 1.24 (1.06, 1.46) | Replaced |

Abbreviations: ATE, arterial thromboembolism; SB, sedentary behavior; PA, physical activity.

*Adjusted covariates include age, race, education, body mass index, Townsend deprivation index, current smoking, current alcohol consumption, depressed mood, hypertension, diabetes, coronary heart disease, and stroke.

All substitutions represent reallocating 1 hour per day from the column behavior to the row behavior.

Figure S4. Associations Between SB, Sleep, LPA, and MVPA Duration and VTE Incidence Among Cancer Patients Stratified by Sex Further Adjusted for Aspirin


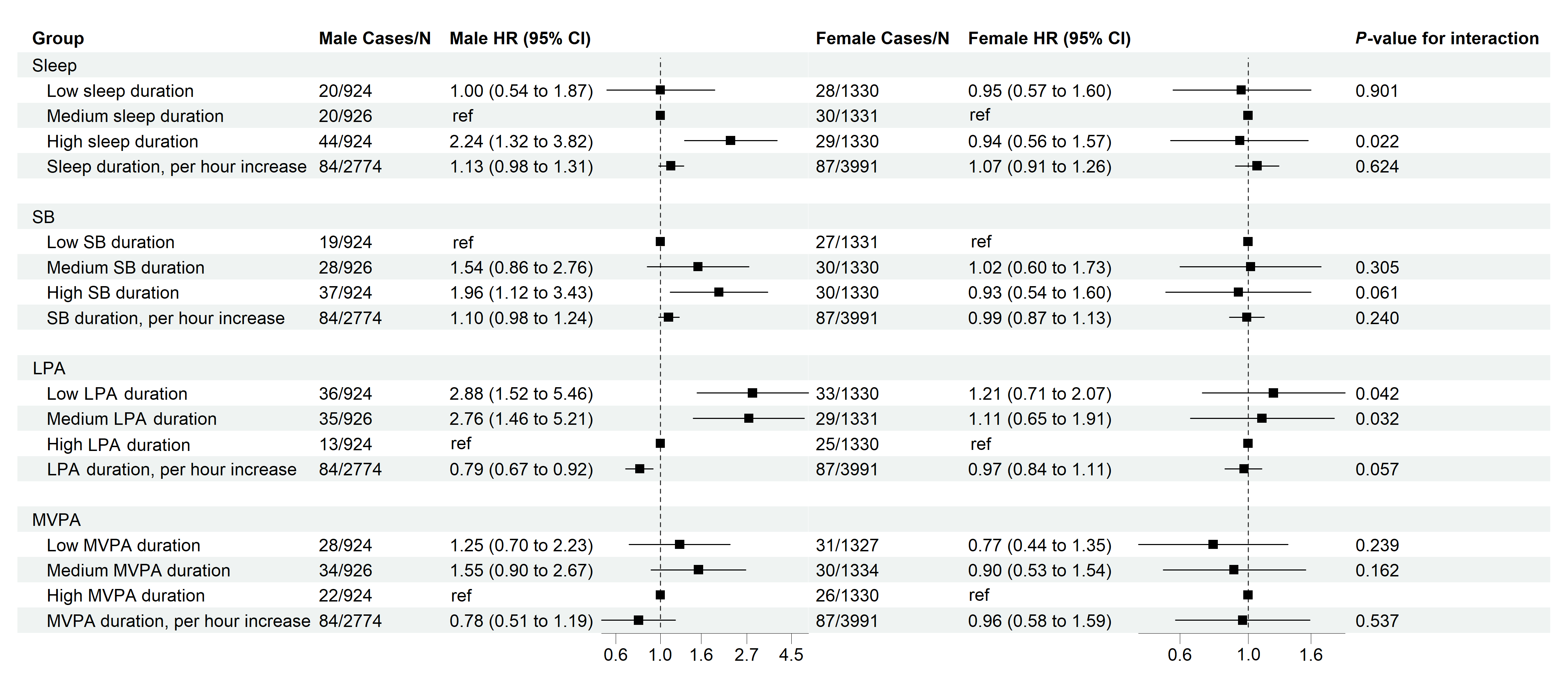
Abbreviations: SB, sedentary behavior; LPA, light physical activity; MVPA, moderate-to-vigorous physical activity; VTE, venous thromboembolism.

Adjusted covariates include aspirin, age, race, education, body mass index, Townsend deprivation index, current smoking, current alcohol consumption, depressed mood, hypertension, diabetes, coronary heart disease, and stroke.

Table S6. Hazard Ratios and 95% Confidence Intervals* for Incident VTE Estimated Using a Multivariable-Adjusted Isotemporal Substitution Cox Regression Model Among Cancer Patients Stratified by Sex (Further Adjusted for Aspirin)

|  | Sleep | SB | LPA | MVPA |
| --- | --- | --- | --- | --- |
| Men (n=2774) |  |  |  |  |
| Replace sleep with | Replaced | 0.94 (0.82, 1.08) | 0.76 (0.64, 0.90) | 0.79 (0.52, 1.20) |
| Replace SB with | 1.06 (0.92, 1.22) | Replaced | 0.81 (0.69, 0.93) | 0.83 (0.56, 1.24) |
| Replace LPA with | 1.32 (1.11, 1.57) | 1.24 (1.07, 1.44) | Replaced | 1.04 (0.67, 1.61) |
| Replace MVPA with | 1.27 (0.84, 1.94) | 1.20 (0.81, 1.79) | 0.97 (0.62, 1.50) | Replaced |
| Women (n=3991) |  |  |  |  |
| Replace sleep with | Replaced | 0.94 (0.77, 1.16) | 0.93 (0.75, 1.16) | 0.92 (0.59, 1.46) |
| Replace SB with | 1.06 (0.86, 1.30) | Replaced | 0.98 (0.82, 1.18) | 0.98 (0.64, 1.50) |
| Replace LPA with | 1.08 (0.87, 1.34) | 1.02 (0.85, 1.22) | Replaced | 1.00 (0.63, 1.58) |
| Replace MVPA with | 1.08 (0.69, 1.71) | 1.02 (0.67, 1.57) | 1.01 (0.63, 1.60) | Replaced |

Abbreviations: VTE, venous thromboembolism; SB, sedentary behavior; LPA, light physical activity; MVPA, moderate-to-vigorous physical activity.

*Adjusted covariates include aspirin, age, race, education, body mass index, Townsend deprivation index, current smoking, current alcohol consumption, depressed mood, hypertension, diabetes, coronary heart disease, and stroke.

All substitutions represent reallocating 1 hour per day from the column behavior to the row behavior.

Figure S5. Associations Between SB, Sleep, LPA, and MVPA Duration and ATE Incidence Among Cancer Patients Stratified by Sex Further Adjusted for Aspirin


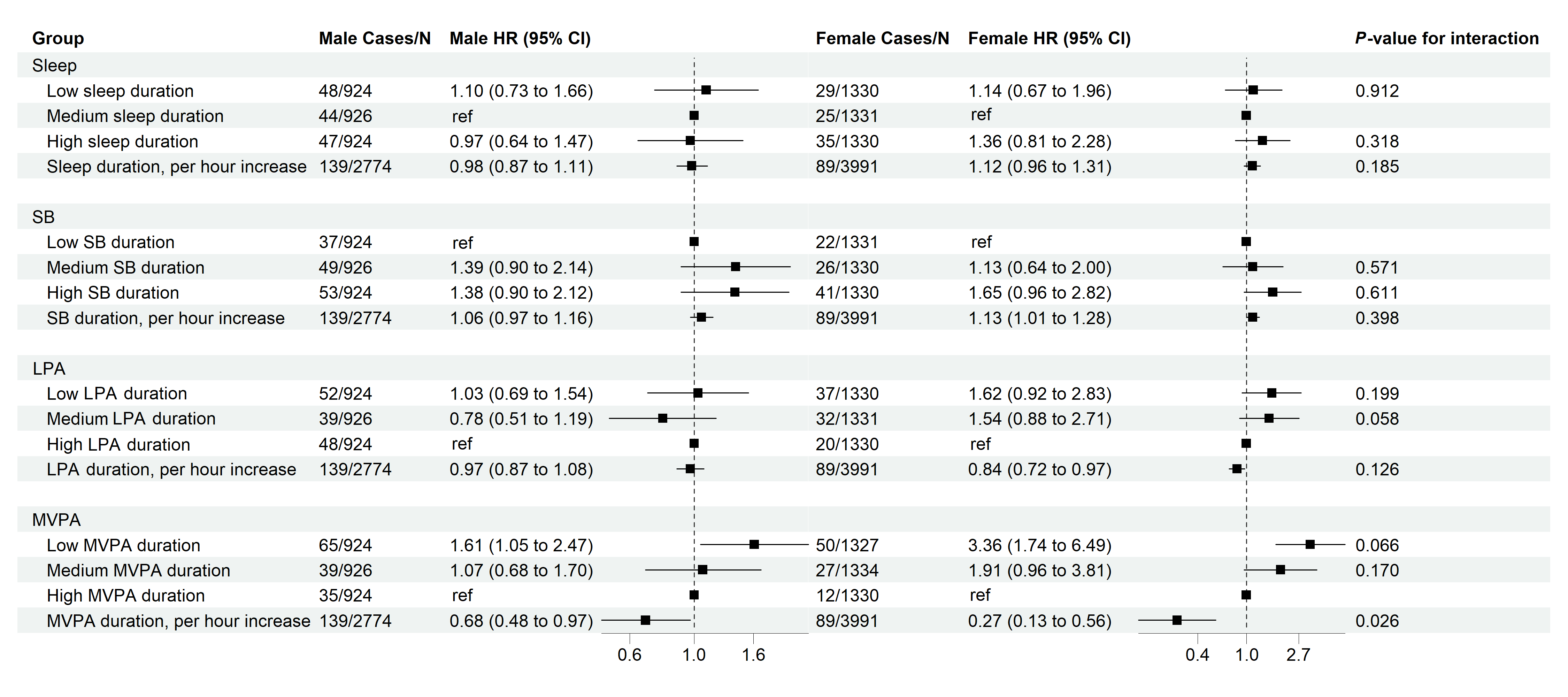
Abbreviations: SB, sedentary behavior; LPA, light physical activity; MVPA, moderate-to-vigorous physical activity; ATE, arterial thromboembolism.

Adjusted covariates include aspirin, age, race, education, body mass index, Townsend deprivation index, current smoking, current alcohol consumption, depressed mood, hypertension, diabetes, coronary heart disease, and stroke.

Table S7. Hazard Ratios and 95% Confidence Intervals* for Incident ATE Estimated Using a Multivariable-Adjusted Isotemporal Substitution Cox Regression Model Among Cancer Patients Stratified by Sex (Further Adjusted for Aspirin)

|  | Sleep | SB | LPA | MVPA |
| --- | --- | --- | --- | --- |
| Men (n=2774) |  |  |  |  |
| Replace sleep with | Replaced | 1.04 (0.91, 1.19) | 1.01 (0.85, 1.19) | 0.71 (0.48, 1.05) |
| Replace SB with | 0.96 (0.84, 1.10) | Replaced | 0.97 (0.86, 1.09) | 0.68 (0.47, 1.00) |
| Replace LPA with | 0.99 (0.84, 1.18) | 1.03 (0.92, 1.16) | Replaced | 0.71 (0.47, 1.05) |
| Replace MVPA with | 1.41 (0.96, 2.08) | 1.46 (1.01, 2.13) | 1.42 (0.95, 2.11) | Replaced |
| Women (n=3991) |  |  |  |  |
| Replace sleep with | Replaced | 0.97 (0.81, 1.16) | 0.84 (0.69, 1.04) | 0.29 (0.14, 0.60) |
| Replace SB with | 1.03 (0.86, 1.23) | Replaced | 0.87 (0.74, 1.01) | 0.30 (0.14, 0.63) |
| Replace LPA with | 1.19 (0.97, 1.46) | 1.15 (0.99, 1.35) | Replaced | 0.34 (0.16, 0.72) |
| Replace MVPA with | 3.47 (1.68, 7.17) | 3.38 (1.59, 7.18) | 2.93 (1.40, 6.14) | Replaced |

Abbreviations: ATE, arterial thromboembolism, SB, sedentary behavior; LPA, light physical activity; MVPA, moderate-to-vigorous physical activity.

*Adjusted covariates include aspirin, age, race, education, body mass index, Townsend deprivation index, current smoking, current alcohol consumption, depressed mood, hypertension, diabetes, coronary heart disease, and stroke.

All substitutions represent reallocating 1 hour per day from the column behavior to the row behavior.

Table S8. Association between Movement Behaviors and VTE Incidence among Cancer Patients Stratified by Sex: Delayed-Entry Cox Models

| Group | Men (n=2774) | | Women (n=3991) | | *P*-value for interaction |
| --- | --- | --- | --- | --- | --- |
|  | Evens/Total | HR (95% CI) | Evens/Total | HR (95% CI) |  |
| Sleep |  |  |  |  |  |
| Low Sleep duration | 20/924 | 1.02 (0.55, 1.91) | 28/1330 | 0.95 (0.56, 1.59) | 0.864 |
| Medium Sleep duration | 20/926 | ref | 30/1331 | ref |  |
| High Sleep duration | 44/924 | 2.34 (1.37, 3.98) | 29/1330 | 0.90 (0.54, 1.51) | 0.012 |
| Sleep duration, per hour increase | 84/2774 | 1.14 (0.98, 1.32) | 87/3991 | 1.06 (0.90, 1.25) | 0.520 |
| SB |  |  |  |  |  |
| Low SB duration | 19/924 | ref | 27/1331 | ref |  |
| Medium SB duration | 28/926 | 1.53 (0.85, 2.74) | 30/1330 | 1.02 (0.60, 1.72) | 0.313 |
| High SB duration | 37/924 | 1.94 (1.11, 3.39) | 30/1330 | 0.91 (0.53, 1.58) | 0.058 |
| SB duration, per hour increase | 84/2774 | 1.10 (0.98, 1.24) | 87/3991 | 0.99 (0.87, 1.12) | 0.232 |
| LIPA |  |  |  |  |  |
| Low LIPA duration | 36/924 | 2.90 (1.53, 5.50) | 33/1330 | 1.17 (0.68, 2.00) | 0.034 |
| Medium LIPA duration | 35/926 | 2.72 (1.44, 5.16) | 29/1331 | 1.10 (0.64, 1.88) | 0.034 |
| High LIPA duration | 13/924 | ref | 25/1330 | ref |  |
| LIPA duration, per hour increase | 84/2774 | 0.79 (0.67, 0.92) | 87/3991 | 0.98 (0.85, 1.13) | 0.047 |
| MVPA |  |  |  |  |  |
| Low MVPA duration | 28/924 | 1.23 (0.69, 2.20) | 31/1327 | 0.76 (0.43, 1.32) | 0.242 |
| Medium MVPA duration | 34/926 | 1.53 (0.89, 2.63) | 30/1334 | 0.89 (0.52, 1.52) | 0.164 |
| High MVPA duration | 22/924 | ref | 26/1330 | ref |  |
| MVPA duration, per hour increase | 84/2774 | 0.78 (0.51, 1.20) | 87/3991 | 0.98 (0.59, 1.64) | 0.502 |

Abbreviations: SB, sedentary behavior; LPA, light physical activity; MVPA, moderate-to-vigorous physical activity; VTE, venous thromboembolism.

Adjusted covariates include age, race, education, body mass index, Townsend deprivation index, current smoking, current alcohol consumption, depressed mood, hypertension, diabetes, coronary heart disease, and stroke.

Table S9. Association between Movement Behaviors and ATE Incidence among Cancer Patients Stratified by Sex: Delayed-Entry Cox Models

| Group | Men (n=2774) | | Women (n=3991) | | *P*-value for interaction |
| --- | --- | --- | --- | --- | --- |
|  | Evens/Total | HR (95% CI) | Evens/Total | HR (95% CI) |  |
| Sleep |  |  |  |  |  |
| Low sleep duration | 48/924 | 1.10 (0.73, 1.65) | 29/1330 | 1.10 (0.64, 1.89) | 1.000 |
| Medium sleep duration | 44/926 | ref | 25/1331 | ref |  |
| High sleep duration | 47/924 | 0.99 (0.65, 1.50) | 35/1330 | 1.31 (0.78, 2.21) | 0.411 |
| Sleep duration, per hour increase | 139/2774 | 0.99 (0.88, 1.12) | 89/3991 | 1.12 (0.96, 1.30) | 0.212 |
| SB |  |  |  |  |  |
| Low SB duration | 37/924 | ref | 22/1331 | ref |  |
| Medium SB duration | 49/926 | 1.33 (0.86, 2.05) | 26/1330 | 1.19 (0.67, 2.11) | 0.762 |
| High SB duration | 53/924 | 1.35 (0.88, 2.07) | 41/1330 | 1.68 (0.98, 2.87) | 0.533 |
| SB duration, per hour increase | 139/2774 | 1.06 (0.96, 1.16) | 89/3991 | 1.12 (1.00, 1.27) | 0.479 |
| LIPA |  |  |  |  |  |
| Low LIPA duration | 52/924 | 1.02 (0.68, 1.52) | 37/1330 | 1.62 (0.92, 2.84) | 0.190 |
| Medium LIPA duration | 39/926 | 0.76 (0.50, 1.16) | 32/1331 | 1.53 (0.87, 2.69) | 0.051 |
| High LIPA duration | 48/924 | ref | 20/1330 | ref |  |
| LIPA duration, per hour increase | 139/2774 | 0.97 (0.87, 1.09) | 89/3991 | 0.84 (0.73, 0.97) | 0.120 |
| MVPA |  |  |  |  |  |
| Low MVPA duration | 65/924 | 1.56 (1.02, 2.40) | 50/1327 | 3.35 (1.74, 6.48) | 0.056 |
| Medium MVPA duration | 39/926 | 1.02 (0.64, 1.62) | 27/1334 | 1.93 (0.97, 3.84) | 0.132 |
| High MVPA duration | 35/924 | ref | 12/1330 | ref |  |
| MVPA duration, per hour increase | 139/2774 | 0.70 (0.49, 0.99) | 89/3991 | 0.28 (0.13, 0.56) | 0.027 |

Abbreviations: SB, sedentary behavior; LPA, light physical activity; MVPA, moderate-to-vigorous physical activity; VTE, venous thromboembolism.

Adjusted covariates include age, race, education, body mass index, Townsend deprivation index, current smoking, current alcohol consumption, depressed mood, hypertension, diabetes, coronary heart disease, and stroke.

Table S10 Hazard Ratios* for Incident VTE Estimated Using Multivariable-Adjusted Delayed-Entry Isotemporal Substitution Cox Regression Models Among Cancer Patients Stratified by Sex

|  | Sleep | SB | LPA | MVPA |
| --- | --- | --- | --- | --- |
| Men (n=2774) |  |  |  |  |
| Replace sleep with | Replaced | 0.94 (0.82, 1.08) | 0.76 (0.64, 0.89) | 0.79 (0.52, 1.20) |
| Replace SB with | 1.06 (0.93, 1.22) | Replaced | 0.81 (0.70, 0.93) | 0.84 (0.56, 1.25) |
| Replace LPA with | 1.32 (1.12, 1.56) | 1.24 (1.07, 1.44) | Replaced | 1.04 (0.67, 1.62) |
| Replace MVPA with | 1.26 (0.83, 1.92) | 1.19 (0.80, 1.78) | 0.96 (0.62, 1.49) | Replaced |
| Women (n=3991) |  |  |  |  |
| Replace sleep with | Replaced | 0.95 (0.77, 1.16) | 0.94 (0.76, 1.16) | 0.95 (0.60, 1.49) |
| Replace SB with | 1.06 (0.87, 1.29) | Replaced | 0.99 (0.83, 1.19) | 1.00 (0.66, 1.53) |
| Replace LPA with | 1.07 (0.86, 1.32) | 1.01 (0.84, 1.20) | Replaced | 1.01 (0.64, 1.60) |
| Replace MVPA with | 1.06 (0.67, 1.66) | 1.00 (0.65, 1.53) | 0.99 (0.63, 1.57) | Replaced |

Abbreviations: SB, sedentary behavior; LPA, light physical activity; MVPA, moderate-to-vigorous physical activity; VTE, venous thromboembolism.

*Adjusted covariates include age, race, education, body mass index, Townsend deprivation index, current smoking, current alcohol consumption, depressed mood, hypertension, diabetes, coronary heart disease, and stroke.

All substitutions represent reallocating 1 hour per day from the column behavior to the row behavior.

Table S11. Hazard Ratios* for Incident ATE Estimated Using Multivariable-Adjusted Delayed-Entry Isotemporal Substitution Cox Regression Models Among Cancer Patients Stratified by Sex

|  | Sleep | SB | LPA | MVPA |
| --- | --- | --- | --- | --- |
| Men (n=2774) |  |  |  |  |
| Replace sleep with | Replaced | 1.03 (0.90, 1.18) | 1.00 (0.85, 1.19) | 0.72 (0.49, 1.06) |
| Replace SB with | 0.97 (0.85, 1.12) | Replaced | 0.97 (0.86, 1.09) | 0.70 (0.48, 1.02) |
| Replace LPA with | 1.00 (0.84, 1.18) | 1.03 (0.91, 1.16) | Replaced | 0.72 (0.48, 1.08) |
| Replace MVPA with | 1.39 (0.94, 2.06) | 1.43 (0.98, 2.10) | 1.39 (0.93, 2.09) | Replaced |
| Women (n=3991) |  |  |  |  |
| Replace sleep with | Replaced | 0.97 (0.82, 1.15) | 0.85 (0.69, 1.04) | 0.29 (0.14, 0.59) |
| Replace SB with | 1.03 (0.87, 1.23) | Replaced | 0.88 (0.76, 1.01) | 0.30 (0.14, 0.62) |
| Replace LPA with | 1.18 (0.97, 1.45) | 1.14 (0.99, 1.33) | Replaced | 0.34 (0.16, 0.70) |
| Replace MVPA with | 3.50 (1.70, 7.19) | 3.38 (1.61, 7.10) | 2.96 (1.42, 6.15) | Replaced |

Abbreviations: SB, sedentary behavior; LPA, light physical activity; MVPA, moderate-to-vigorous physical activity; VTE, venous thromboembolism.

*Adjusted covariates include age, race, education, body mass index, Townsend deprivation index, current smoking, current alcohol consumption, depressed mood, hypertension, diabetes, coronary heart disease, and stroke.

All substitutions represent reallocating 1 hour per day from the column behavior to the row behavior.

Table S12. Association between Movement Behaviors and VTE Incidence among Cancer Patients Stratified by Sex and Cancer Diagnosis to Baseline Duration (≤5 years): Delayed-Entry Cox Models

| Group | Men (n=1270) | | Women (n=1311) | | *P*-value for interaction |
| --- | --- | --- | --- | --- | --- |
|  | Evens/Total | HR (95% CI) | Evens/Total | HR (95% CI) |  |
| Sleep |  |  |  |  |  |
| Low Sleep duration | 8/406 | 0.94 (0.36, 2.45) | 13/434 | 1.10 (0.48, 2.55) | 0.809 |
| Medium Sleep duration | 9/431 | ref | 10/417 | ref |  |
| High Sleep duration | 24/433 | 2.50 (1.15, 5.42) | 14/460 | 1.13 (0.50, 2.57) | 0.167 |
| Sleep duration, per hour increase | 41/1270 | 1.11 (0.91, 1.36) | 37/1311 | 1.11 (0.88, 1.40) | 1.000 |
| SB |  |  |  |  |  |
| Low SB duration | 9/440 | ref | 11/413 | ref |  |
| Medium SB duration | 17/415 | 2.16 (0.96, 4.87) | 11/447 | 0.83 (0.36, 1.94) | 0.109 |
| High SB duration | 15/415 | 1.82 (0.79, 4.21) | 15/451 | 0.93 (0.41, 1.22) | 0.188 |
| SB duration, per hour increase | 41/1270 | 1.09 (0.92, 1.29) | 37/1311 | 1.04 (0.85, 1.26) | 0.723 |
| LIPA |  |  |  |  |  |
| Low LIPA duration | 19/425 | 3.82 (1.41, 10.34) | 17/469 | 1.56 (0.66, 3.69) | 0.182 |
| Medium LIPA duration | 17/420 | 3.47 (1.28, 9.42) | 12/423 | 1.34 (0.55, 3.30) | 0.168 |
| High LIPA duration | 5/425 | ref | 8/419 | ref |  |
| LIPA duration, per hour increase | 41/1270 | 0.77 (0.61, 0.97) | 37/1311 | 0.89 (0.72, 1.11) | 0.371 |
| MVPA |  |  |  |  |  |
| Low MVPA duration | 14/402 | 0.95 (0.43, 2.08) | 17/432 | 0.99 (0.44, 2.24) | 0.943 |
| Medium MVPA duration | 13/423 | 0.96 (0.45, 2.07) | 9/463 | 0.60 (0.24, 1.46) | 0.436 |
| High MVPA duration | 14/445 | ref | 11/416 | ref |  |
| MVPA duration, per hour increase | 41/1270 | 0.98 (0.55, 1.73) | 37/1311 | 0.91 (0.40, 2.09) | 0.885 |

Abbreviations: SB, sedentary behavior; LPA, light physical activity; MVPA, moderate-to-vigorous physical activity; VTE, venous thromboembolism.

Adjusted covariates include age, race, education, body mass index, Townsend deprivation index, current smoking, current alcohol consumption, depressed mood, hypertension, diabetes, coronary heart disease, and stroke.

Table S13. Association between Movement Behaviors and ATE Incidence among Cancer Patients Stratified by Sex and Cancer Diagnosis to Baseline Duration (≤5 years): Delayed-Entry Cox Models

| Group | Men (n=1270) | | Women (n=1311) | | *P*-value for interaction |
| --- | --- | --- | --- | --- | --- |
|  | Evens/Total | HR (95% CI) | Evens/Total | HR (95% CI) |  |
| Sleep |  |  |  |  |  |
| Low sleep duration | 21/406 | 1.30 (0.68, 2.51) | 12/434 | 1.40 (0.56, 3.47) | 0.897 |
| Medium sleep duration | 16/431 | ref | 8/417 | ref |  |
| High sleep duration | 21/433 | 1.14 (0.59, 2.20) | 12/460 | 1.34 (0.54, 3.34) | 0.778 |
| Sleep duration, per hour increase | 58/1270 | 1.01 (0.84, 1.22) | 32/1311 | 1.15 (0.89, 1.48) | 0.420 |
| SB |  |  |  |  |  |
| Low SB duration | 17/440 | ref | 11/413 | ref |  |
| Medium SB duration | 20/415 | 1.26 (0.65, 2.44) | 6/447 | 0.49 (0.18, 1.33) | 0.123 |
| High SB duration | 21/415 | 1.22 (0.63, 2.35) | 15/451 | 1.07 (0.47, 2.45) | 0.808 |
| SB duration, per hour increase | 58/1270 | 1.06 (0.92, 1.22) | 32/1311 | 0.98 (0.80, 1.21) | 0.539 |
| LIPA |  |  |  |  |  |
| Low LIPA duration | 24/425 | 1.18 (0.64, 2.19) | 11/469 | 0.76 (0.32, 1.83) | 0.419 |
| Medium LIPA duration | 15/420 | 0.82 (0.42, 1.62) | 10/423 | 0.87 (0.37, 2.07) | 0.916 |
| High LIPA duration | 19/425 | ref | 11/419 | ref |  |
| LIPA duration, per hour increase | 58/1270 | 0.93 (0.78, 1.11) | 32/1311 | 0.98 (0.79, 1.23) | 0.717 |
| MVPA |  |  |  |  |  |
| Low MVPA duration | 24/402 | 1.23 (0.65, 2.30) | 15/432 | 4.62 (1.28, 16.72) | 0.070 |
| Medium MVPA duration | 15/423 | 0.67 (0.33, 1.37) | 14/463 | 4.28 (1.22, 15.06) | 0.012 |
| High MVPA duration | 19/445 | ref | 3/416 | ref |  |
| MVPA duration, per hour increase | 58/1270 | 0.87 (0.52, 1.45) | 32/1311 | 0.28 (0.08, 0.92) | 0.093 |

Abbreviations: SB, sedentary behavior; LPA, light physical activity; MVPA, moderate-to-vigorous physical activity; VTE, venous thromboembolism.

Adjusted covariates include age, race, education, body mass index, Townsend deprivation index, current smoking, current alcohol consumption, depressed mood, hypertension, diabetes, coronary heart disease, and stroke.

Table S14. Association between Movement Behaviors and VTE Incidence among Cancer Patients Stratified by Sex and Cancer Diagnosis to Baseline Duration (>5 years): Delayed-Entry Cox Models

| Group | Men (n=1504) | | Women (n=2680) | | *P*-value for interaction |
| --- | --- | --- | --- | --- | --- |
|  | Evens/Total | HR (95% CI) | Evens/Total | HR (95% CI) |  |
| Sleep |  |  |  |  |  |
| Low Sleep duration | 12/518 | 1.01 (0.44, 2.30) | 15/896 | 0.79 (0.40, 1.55) | 0.652 |
| Medium Sleep duration | 11/495 | ref | 20/914 | ref |  |
| High Sleep duration | 20/491 | 2.20 (0.96, 4.25) | 15/870 | 0.73 (0.37, 1.43) | 0.032 |
| Sleep duration, per hour increase | 43/1504 | 1.14 (0.92, 1.41) | 50/2680 | 1.04 (0.83, 1.30) | 0.561 |
| SB |  |  |  |  |  |
| Low SB duration | 10/484 | ref | 16/918 | ref |  |
| Medium SB duration | 11/511 | 1.07 (0.45, 2.53) | 19/883 | 1.14 (0.58, 2.25) | 0.910 |
| High SB duration | 22/509 | 2.08 (0.98, 4.45) | 15/879 | 0.85 (0.40, 1.78) | 0.099 |
| SB duration, per hour increase | 43/1504 | 1.12 (0.95, 1.32) | 50/2680 | 0.94 (0.79, 1.11) | 0.147 |
| LIPA |  |  |  |  |  |
| Low LIPA duration | 17/499 | 2.35 (1.01, 5.50) | 16/861 | 0.99 (0.49, 2.01) | 0.124 |
| Medium LIPA duration | 18/506 | 2.23 (0.96, 5.15) | 17/908 | 1.01 (0.51, 1.99) | 0.151 |
| High LIPA duration | 8/499 | ref | 17/911 | ref |  |
| LIPA duration, per hour increase | 43/1504 | 0.79 (0.64, 0.98) | 50/2680 | 1.05 (0.87, 1.26) | 0.048 |
| MVPA |  |  |  |  |  |
| Low MVPA duration | 14/522 | 1.71 (0.69, 4.21) | 14/895 | 0.58 (0.26, 1.29) | 0.079 |
| Medium MVPA duration | 21/503 | 2.41 (1.05, 5.52) | 21/871 | 1.05 (0.53, 2.10) | 0.131 |
| High MVPA duration | 8/479 | ref | 15/914 | ref |  |
| MVPA duration, per hour increase | 43/1504 | 0.61 (0.32, 1.17) | 50/2680 | 1.06 (0.55, 2.05) | 0.241 |

Abbreviations: SB, sedentary behavior; LPA, light physical activity; MVPA, moderate-to-vigorous physical activity; VTE, venous thromboembolism.

Adjusted covariates include age, race, education, body mass index, Townsend deprivation index, current smoking, current alcohol consumption, depressed mood, hypertension, diabetes, coronary heart disease, and stroke.

Table S15. Association between Movement Behaviors and ATE Incidence among Cancer Patients Stratified by Sex and Cancer Diagnosis to Baseline Duration (>5 years): Delayed-Entry Cox Models

| Group | Men (n=1504) | | Women (n=2680) | | *P*-value for interaction |
| --- | --- | --- | --- | --- | --- |
|  | Evens/Total | HR (95% CI) | Evens/Total | HR (95% CI) |  |
| Sleep |  |  |  |  |  |
| Low sleep duration | 27/518 | 0.98 (0.58, 1.67) | 17/896 | 0.98 (0.50, 1.92) | 1.000 |
| Medium sleep duration | 28/495 | ref | 17/914 | ref |  |
| High sleep duration | 36/491 | 0.92 (0.54, 1.58) | 23/870 | 1.30 (0.69, 2.46) | 0.415 |
| Sleep duration, per hour increase | 81/1504 | 0.98 (0.83, 1.15) | 57/2680 | 1.09 (0.89, 1.33) | 0.420 |
| SB |  |  |  |  |  |
| Low SB duration | 20/484 | ref | 11/918 | ref |  |
| Medium SB duration | 29/511 | 1.31 (0.74, 2.35) | 20/883 | 1.97 (0.94, 4.16) | 0.396 |
| High SB duration | 32/509 | 1.50 (0.85, 2.65) | 26/879 | 2.26 (1.09, 4.68) | 0.385 |
| SB duration, per hour increase | 81/1504 | 1.07 (0.95, 1.21) | 57/2680 | 1.19 (1.03, 1.38) | 0.272 |
| LIPA |  |  |  |  |  |
| Low LIPA duration | 28/499 | 0.98 (0.58, 1.65) | 26/861 | 2.67 (1.22, 5.82) | 0.037 |
| Medium LIPA duration | 24/506 | 0.76 (0.44, 1.31) | 22/908 | 2.32 (1.06, 5.09) | 0.022 |
| High LIPA duration | 29/499 | ref | 9/911 | ref |  |
| LIPA duration, per hour increase | 81/1504 | 0.99 (0.85, 1.15) | 57/2680 | 0.77 (0.64, 0.93) | 0.040 |
| MVPA |  |  |  |  |  |
| Low MVPA duration | 39/522 | 2.06 (1.13, 3.76) | 35/895 | 2.92 (1.35, 6.35) | 0.485 |
| Medium MVPA duration | 26/503 | 1.45 (0.77, 2.73) | 13/871 | 1.18 (0.49, 2.80) | 0.708 |
| High MVPA duration | 16/479 | ref | 9/914 | ref |  |
| MVPA duration, per hour increase | 81/1504 | 0.56 (0.34, 0.90) | 57/2680 | 0.28 (0.11, 0.69) | 0.191 |

Abbreviations: SB, sedentary behavior; LPA, light physical activity; MVPA, moderate-to-vigorous physical activity; VTE, venous thromboembolism.

Adjusted covariates include age, race, education, body mass index, Townsend deprivation index, current smoking, current alcohol consumption, depressed mood, hypertension, diabetes, coronary heart disease, and stroke.

Table S16. Association between SB, Sleep, LPA, and MVPA Duration and Incident VTE Among Cancer Patients Stratified by Sex: Competing-Risk Models

| Group | Men (n=2774) | | Women (n=3991) | | *P*-value for interaction |
| --- | --- | --- | --- | --- | --- |
|  | Evens/Total | HR (95% CI) | Evens/Total | HR (95% CI) |  |
| Sleep |  |  |  |  |  |
| Low Sleep duration | 20/924 | 1.00 (0.54, 1.85) | 28/1330 | 0.95 (0.57, 1.59) | 0.900 |
| Medium Sleep duration | 20/926 | ref | 30/1331 | ref |  |
| High Sleep duration | 44/924 | 2.18 (1.29, 3.70) | 29/1330 | 0.93 (0.55, 1.56) | 0.024 |
| Sleep duration, per hour increase | 84/2774 | 1.12 (0.98, 1.27) | 87/3991 | 1.06 (0.88, 1.28) | 0.636 |
| SB |  |  |  |  |  |
| Low SB duration | 19/924 | ref | 27/1331 | ref |  |
| Medium SB duration | 28/926 | 1.54 (0.87, 2.75) | 30/1330 | 1.02 (0.60, 1.73) | 0.302 |
| High SB duration | 37/924 | 1.92 (1.10, 3.34) | 30/1330 | 0.90 (0.52, 1.58) | 0.089 |
| SB duration, per hour increase | 84/2774 | 1.10 (0.99, 1.21) | 87/3991 | 0.99 (0.85, 1.15) | 0.255 |
| LIPA |  |  |  |  |  |
| Low LIPA duration | 36/924 | 2.78 (1.47, 5.24) | 33/1330 | 1.17 (0.68, 2.02) | 0.043 |
| Medium LIPA duration | 35/926 | 2.72 (1.44, 5.14) | 29/1331 | 1.09 (0.64, 1.87) | 0.031 |
| High LIPA duration | 13/924 | ref | 25/1330 | ref |  |
| LIPA duration, per hour increase | 84/2774 | 0.80 (0.70, 0.92) | 87/3991 | 0.98 (0.83, 1.16) | 0.066 |
| MVPA |  |  |  |  |  |
| Low MVPA duration | 28/924 | 1.20 (0.66, 2.16) | 31/1327 | 0.75 (0.44, 1.29) | 0.250 |
| Medium MVPA duration | 34/926 | 1.52 (0.88, 2.63) | 30/1334 | 0.90 (0.53, 1.53) | 0.178 |
| High MVPA duration | 22/924 | ref | 26/1330 | ref |  |
| MVPA duration, per hour increase | 84/2774 | 0.81 (0.54, 1.21) | 87/3991 | 0.98 (0.64, 1.49) | 0.523 |

Abbreviations: SB, sedentary behavior; LPA, light physical activity; MVPA, moderate-to-vigorous physical activity; VTE, venous thromboembolism.

Adjusted covariates include age, race, education, body mass index, Townsend deprivation index, current smoking, current alcohol consumption, depressed mood, hypertension, diabetes, coronary heart disease, and stroke.

Table S17. Association between SB, Sleep, LPA, and MVPA Duration and Incident ATE Among Cancer Patients Stratified by Sex: Competing-Risk Models

| Group | Men (n=2774) | | Women (n=3991) | | *P*-value for interaction |
| --- | --- | --- | --- | --- | --- |
|  | Evens/Total | HR (95% CI) | Evens/Total | HR (95% CI) |  |
| Sleep |  |  |  |  |  |
| Low sleep duration | 48/924 | 1.10 (0.73, 1.65) | 29/1330 | 1.15 (0.67, 1.96) | 0.897 |
| Medium sleep duration | 44/926 | ref | 25/1331 | ref |  |
| High sleep duration | 47/924 | 0.95 (0.63, 1.45) | 35/1330 | 1.33 (0.80, 2.23) | 0.318 |
| Sleep duration, per hour increase | 139/2774 | 0.98 (0.85, 1.12) | 89/3991 | 1.11 (0.93, 1.33) | 0.280 |
| SB |  |  |  |  |  |
| Low SB duration | 37/924 | ref | 22/1331 | ref |  |
| Medium SB duration | 49/926 | 1.36 (0.88, 2.08) | 26/1330 | 1.14 (0.64, 2.03) | 0.631 |
| High SB duration | 53/924 | 1.32 (0.87, 2.02) | 41/1330 | 1.62 (0.92, 2.86) | 0.570 |
| SB duration, per hour increase | 139/2774 | 1.05 (0.96, 1.16) | 89/3991 | 1.12 (0.99, 1.28) | 0.428 |
| LIPA |  |  |  |  |  |
| Low LIPA duration | 52/924 | 0.99 (0.66, 1.48) | 37/1330 | 1.57 (0.88, 2.80) | 0.200 |
| Medium LIPA duration | 39/926 | 0.76 (0.50, 1.16) | 32/1331 | 1.52 (0.85, 2.69) | 0.057 |
| High LIPA duration | 48/924 | ref | 20/1330 | ref |  |
| LIPA duration, per hour increase | 139/2774 | 0.98 (0.87, 1.11) | 89/3991 | 0.85 (0.72, 0.99) | 0.164 |
| MVPA |  |  |  |  |  |
| Low MVPA duration | 65/924 | 1.49 (0.96, 2.31) | 50/1327 | 3.25 (1.72, 6.15) | 0.048 |
| Medium MVPA duration | 39/926 | 1.03 (0.65, 1.63) | 27/1334 | 1.92 (0.97, 3.81) | 0.139 |
| High MVPA duration | 35/924 | ref | 12/1330 | ref |  |
| MVPA duration, per hour increase | 139/2774 | 0.73 (0.50, 1.06) | 89/3991 | 0.29 (0.13, 0.61) | 0.035 |

Abbreviations: SB, sedentary behavior; LPA, light physical activity; MVPA, moderate-to-vigorous physical activity; VTE, venous thromboembolism.

Adjusted covariates include age, race, education, body mass index, Townsend deprivation index, current smoking, current alcohol consumption, depressed mood, hypertension, diabetes, coronary heart disease, and stroke.

Table S18 Hazard Ratios* for Incident VTE Estimated Using Multivariable-Adjusted Isotemporal Substitution Competing-Risk Models Among Cancer Patients Stratified by Sex

|  | Sleep | SB | LPA | MVPA |
| --- | --- | --- | --- | --- |
| Men (n=2774) |  |  |  |  |
| Replace sleep with | Replaced | 0.96 (0.84, 1.09) | 0.78 (0.66, 0.92) | 0.82 (0.54, 1.25) |
| Replace SB with | 1.05 (0.92, 1.20) | Replaced | 0.82 (0.71, 0.95) | 0.86 (0.58, 1.28) |
| Replace LPA with | 1.28 (1.08, 1.51) | 1.22 (1.06, 1.41) | Replaced | 1.05 (0.68, 1.63) |
| Replace MVPA with | 1.21 (0.80, 1.84) | 1.16 (0.78, 1.72) | 0.95 (0.61, 1.47) | Replaced |
| Women (n=3991) |  |  |  |  |
| Replace sleep with | Replaced | 0.95 (0.77, 1.16) | 0.94 (0.76, 1.17) | 0.94 (0.60, 1.48) |
| Replace SB with | 1.06 (0.86, 1.30) | Replaced | 0.99 (0.83, 1.19) | 1.00 (0.65, 1.52) |
| Replace LPA with | 1.07 (0.86, 1.32) | 1.01 (0.84, 1.21) | Replaced | 1.00 (0.63, 1.59) |
| Replace MVPA with | 1.06 (0.68, 1.67) | 1.01 (0.66, 1.53) | 1.00 (0.63, 1.58) | Replaced |

Abbreviations: SB, sedentary behavior; LPA, light physical activity; MVPA, moderate-to-vigorous physical activity; VTE, venous thromboembolism.

*Adjusted covariates include age, race, education, body mass index, Townsend deprivation index, current smoking, current alcohol consumption, depressed mood, hypertension, diabetes, coronary heart disease, and stroke.

All substitutions represent reallocating 1 hour per day from the column behavior to the row behavior.

Table S19. Hazard Ratios* for Incident ATE Estimated Using Multivariable-Adjusted Isotemporal Substitution Competing-Risk Models Among Cancer Patients Stratified by Sex

|  | Sleep | SB | LPA | MVPA |
| --- | --- | --- | --- | --- |
| Men (n=2774) |  |  |  |  |
| Replace sleep with | Replaced | 1.04 (0.91, 1.19) | 1.02 (0.86, 1.20) | 0.76 (0.51, 1.11) |
| Replace SB with | 0.96 (0.84, 1.10) | Replaced | 0.98 (0.87, 1.10) | 0.73 (0.50, 1.05) |
| Replace LPA with | 0.98 (0.83, 1.16) | 1.02 (0.91, 1.15) | Replaced | 0.74 (0.50, 1.10) |
| Replace MVPA with | 1.33 (0.90, 1.95) | 1.38 (0.95, 2.00) | 1.35 (0.91, 2.01) | Replaced |
| Women (n=3991) |  |  |  |  |
| Replace sleep with | Replaced | 0.97 (0.82, 1.16) | 0.85 (0.70, 1.05) | 0.30 (0.15, 0.61) |
| Replace SB with | 1.03 (0.86, 1.23) | Replaced | 0.88 (0.75, 1.02) | 0.31 (0.15, 0.65) |
| Replace LPA with | 1.17 (0.96, 1.44) | 1.14 (0.98, 1.33) | Replaced | 0.35 (0.17, 0.73) |
| Replace MVPA with | 3.34 (1.63, 6.85) | 3.25 (1.54, 6.85) | 2.86 (1.37, 5.95) | Replaced |

Abbreviations: SB, sedentary behavior; LPA, light physical activity; MVPA, moderate-to-vigorous physical activity; VTE, venous thromboembolism.

*Adjusted covariates include age, race, education, body mass index, Townsend deprivation index, current smoking, current alcohol consumption, depressed mood, hypertension, diabetes, coronary heart disease, and stroke.

All substitutions represent reallocating 1 hour per day from the column behavior to the row behavior.

Table S20. Association between SB, Sleep, LPA, and MVPA Duration and VTE Incidence Among Cancer Patients Stratified by Sex Using Tertiles Derived from the Overall Cancer Sample

| Group | Men (n=2774) | | Women (n=3991) | | *P*-value for interaction |
| --- | --- | --- | --- | --- | --- |
|  | Evens/Total | HR (95% CI) | Evens/Total | HR (95% CI) |  |
| Sleep |  |  |  |  |  |
| Low Sleep duration | 23/962 | 1.24 (0.66, 2.33) | 28/1292 | 1.01 (0.60, 1.69) | 0.622 |
| Medium Sleep duration | 17/882 | ref | 30/1375 | ref |  |
| High Sleep duration | 44/930 | 2.50 (1.42, 4.38) | 29/1324 | 0.97 (0.58, 1.62) | 0.015 |
| Sleep duration, per hour increase | 84/2774 | 1.12 (0.98, 1.27) | 87/3991 | 1.06 (0.88, 1.28) | 0.636 |
| SB |  |  |  |  |  |
| Low SB duration | 15/744 | ref | 32/1509 | ref |  |
| Medium SB duration | 24/882 | 1.40 (0.73, 2.68) | 29/1376 | 0.92 (0.56, 1.53) | 0.317 |
| High SB duration | 45/1148 | 2.00 (1.11, 3.61) | 26/1106 | 0.91 (0.53, 1.56) | 0.054 |
| SB duration, per hour increase | 84/2774 | 1.10 (0.99, 1.21) | 87/3991 | 0.99 (0.85, 1.15) | 0.255 |
| LIPA |  |  |  |  |  |
| Low LIPA duration | 49/1236 | 2.89 (1.42, 5.91) | 26/1020 | 1.22 (0.71, 2.10) | 0.059 |
| Medium LIPA duration | 26/909 | 2.07 (0.97, 4.42) | 30/1346 | 1.09 (0.66, 1.81) | 0.168 |
| High LIPA duration | 9/629 | ref | 31/1625 | ref |  |
| LIPA duration, per hour increase | 84/2774 | 0.80 (0.70, 0.92) | 87/3991 | 0.98 (0.83, 1.16) | 0.066 |
| MVPA |  |  |  |  |  |
| Low MVPA duration | 23/666 | 1.28 (0.73, 2.26） | 35/1594 | 0.75 (0.42, 1.35) | 0.197 |
| Medium MVPA duration | 30/937 | 1.22 (0.74, 2.03) | 32/1317 | 1.04 (0.59, 1.83) | 0.680 |
| High MVPA duration | 31/1171 | ref | 20/1080 | ref |  |
| MVPA duration, per hour increase | 84/2774 | 0.81 (0.54, 1.21) | 87/3991 | 0.98 (0.64, 1.49) | 0.523 |

Abbreviations: SB, sedentary behavior; LPA, light physical activity; MVPA, moderate-to-vigorous physical activity; VTE, venous thromboembolism.

Adjusted covariates include age, race, education, body mass index, Townsend deprivation index, current smoking, current alcohol consumption, depressed mood, hypertension, diabetes, coronary heart disease, and stroke.

Table S21. Association between SB, Sleep, LPA, and MVPA Duration and ATE Incidence Among Cancer Patients Stratified by Sex Using Tertiles Derived from the Overall Cancer Sample

| Group | Men (n=2774) | | Women (n=3991) | | *P*-value for interaction |
| --- | --- | --- | --- | --- | --- |
|  | Evens/Total | HR (95% CI) | Evens/Total | HR (95% CI) |  |
| Sleep |  |  |  |  |  |
| Low sleep duration | 48/962 | 1.00 (0.67, 1.51) | 28/1292 | 1.13 (0.66, 1.93) | 0.722 |
| Medium sleep duration | 44/882 | ref | 26/1375 | ref |  |
| High sleep duration | 47/930 | 0.94 (0.62, 1.41) | 35/1324 | 1.34 (0.80, 2.24) | 0.291 |
| Sleep duration, per hour increase | 139/2774 | 0.98 (0.85, 1.12) | 89/3991 | 1.11 (0.93, 1.33) | 0.280 |
| SB |  |  |  |  |  |
| Low SB duration | 33/744 | ref | 24/1509 | ref |  |
| Medium SB duration | 39/882 | 1.01 (0.63, 1.61) | 28/1376 | 1.24 (0.71, 2.14) | 0.579 |
| High SB duration | 67/1148 | 1.29 (0.85, 1.97) | 37/1106 | 1.88 (1.10, 3.21) | 0.278 |
| SB duration, per hour increase | 139/2774 | 1.05 (0.96, 1.16) | 89/3991 | 1.12 (0.99, 1.28) | 0.428 |
| LIPA |  |  |  |  |  |
| Low LIPA duration | 62/1236 | 0.84 (0.56, 1.28) | 30/1020 | 1.63 (0.95, 2.82) | 0.057 |
| Medium LIPA duration | 41/909 | 0.77 (0.49, 1.21) | 33/1346 | 1.43 (0.85, 2.40) | 0.078 |
| High LIPA duration | 36/629 | ref | 26/1625 | ref |  |
| LIPA duration, per hour increase | 139/2774 | 0.98 (0.87, 1.11) | 89/3991 | 0.85 (0.72, 0.99) | 0.164 |
| MVPA |  |  |  |  |  |
| Low MVPA duration | 51/666 | 1.67 (1.09, 2.56) | 57/1594 | 3.82 (1.78, 8.18) | 0.064 |
| Medium MVPA duration | 43/937 | 1.15 (0.76, 1.76) | 24/1317 | 2.23 (1.00, 4.99) | 0.152 |
| High MVPA duration | 45/1171 | ref | 8/1080 | ref |  |
| MVPA duration, per hour increase | 139/2774 | 0.73 (0.50, 1.06) | 89/3991 | 0.29 (0.13, 0.61) | 0.035 |

Abbreviations: SB, sedentary behavior; LPA, light physical activity; MVPA, moderate-to-vigorous physical activity; VTE, venous thromboembolism.

Adjusted covariates include age, race, education, body mass index, Townsend deprivation index, current smoking, current alcohol consumption, depressed mood, hypertension, diabetes, coronary heart disease, and stroke.

Table S22 Hazard Ratios* for Incident VTE Estimated Using a Multivariable-Adjusted Isotemporal Substitution Cox Regression Model (30 min/day Reallocation) Among Cancer Patients Stratified by Sex

|  | Sleep | SB | LPA | MVPA |
| --- | --- | --- | --- | --- |
| Men (n=2774) |  |  |  |  |
| Replace sleep with | Replaced | 0.97 (0.91, 1.04) | 0.87 (0.80, 0.95) | 0.89 (0.72, 1.09) |
| Replace SB with | 1.03 (0.96, 1.10) | Replaced | 0.90 (0.83, 0.97) | 0.91 (0.75, 1.11) |
| Replace LPA with | 1.15 (1.05, 1.25) | 1.12 (1.04, 1.20) | Replaced | 1.02 (0.82, 1.27) |
| Replace MVPA with | 1.13 (0.92, 1.39) | 1.10 (0.90, 1.34) | 0.98 (0.79, 1.23) | Replaced |
| Women (n=3991) |  |  |  |  |
| Replace sleep with | Replaced | 0.97 (0.88, 1.08) | 0.96 (0.86, 1.08) | 0.96 (0.76, 1.20) |
| Replace SB with | 1.03 (0.93, 1.14) | Replaced | 0.99 (0.91, 1.09) | 0.99 (0.80, 1.22) |
| Replace LPA with | 1.04 (0.93, 1.16) | 1.01 (0.92, 1.10) | Replaced | 1.00 (0.79, 1.25) |
| Replace MVPA with | 1.04 (0.84, 1.31) | 1.01 (0.82, 1.25) | 1.00 (0.80, 1.26) | Replaced |

Abbreviations: SB, sedentary behavior; LPA, light physical activity; MVPA, moderate-to-vigorous physical activity; VTE, venous thromboembolism.

*Adjusted covariates include age, race, education, body mass index, Townsend deprivation index, current smoking, current alcohol consumption, depressed mood, hypertension, diabetes, coronary heart disease, and stroke.

All substitutions represent reallocating 30 min per day from the column behavior to the row behavior.

Table S23. Hazard Ratios* for Incident ATE Estimated Using a Multivariable-Adjusted Isotemporal Substitution Cox Regression Model (30 min/day Reallocation) Among Cancer Patients Stratified by Sex

|  | Sleep | SB | LPA | MVPA |
| --- | --- | --- | --- | --- |
| Men (n=2774) |  |  |  |  |
| Replace sleep with | Replaced | 1.02 (0.95, 1.09) | 1.00 (0.92, 1.09) | 0.84 (0.69, 1.03) |
| Replace SB with | 0.98 (0.92, 1.06) | Replaced | 0.99 (0.93, 1.05) | 0.83 (0.69, 1.01) |
| Replace LPA with | 1.00 (0.92, 1.09) | 1.02 (0.96, 1.08) | Replaced | 0.84 (0.69, 1.03) |
| Replace MVPA with | 1.19 (0.97, 1.44) | 1.20 (0.99, 1.45) | 1.19 (0.97, 1.45) | Replaced |
| Women (n=3991) |  |  |  |  |
| Replace sleep with | Replaced | 0.99 (0.90, 1.08) | 0.92 (0.83, 1.02) | 0.54 (0.38, 0.77) |
| Replace SB with | 1.01 (0.93, 1.11) | Replaced | 0.93 (0.86, 1.01) | 0.55 (0.38, 0.79) |
| Replace LPA with | 1.09 (0.98, 1.21) | 1.07 (0.99, 1.16) | Replaced | 0.59 (0.41, 0.85) |
| Replace MVPA with | 1.86 (1.30, 2.66) | 1.83 (1.26, 2.67) | 1.71 (1.18, 2.47) | Replaced |

Abbreviations: SB, sedentary behavior; LPA, light physical activity; MVPA, moderate-to-vigorous physical activity; VTE, venous thromboembolism.

*Adjusted covariates include age, race, education, body mass index, Townsend deprivation index, current smoking, current alcohol consumption, depressed mood, hypertension, diabetes, coronary heart disease, and stroke.

All substitutions represent reallocating 30 min per day from the column behavior to the row behavior.
